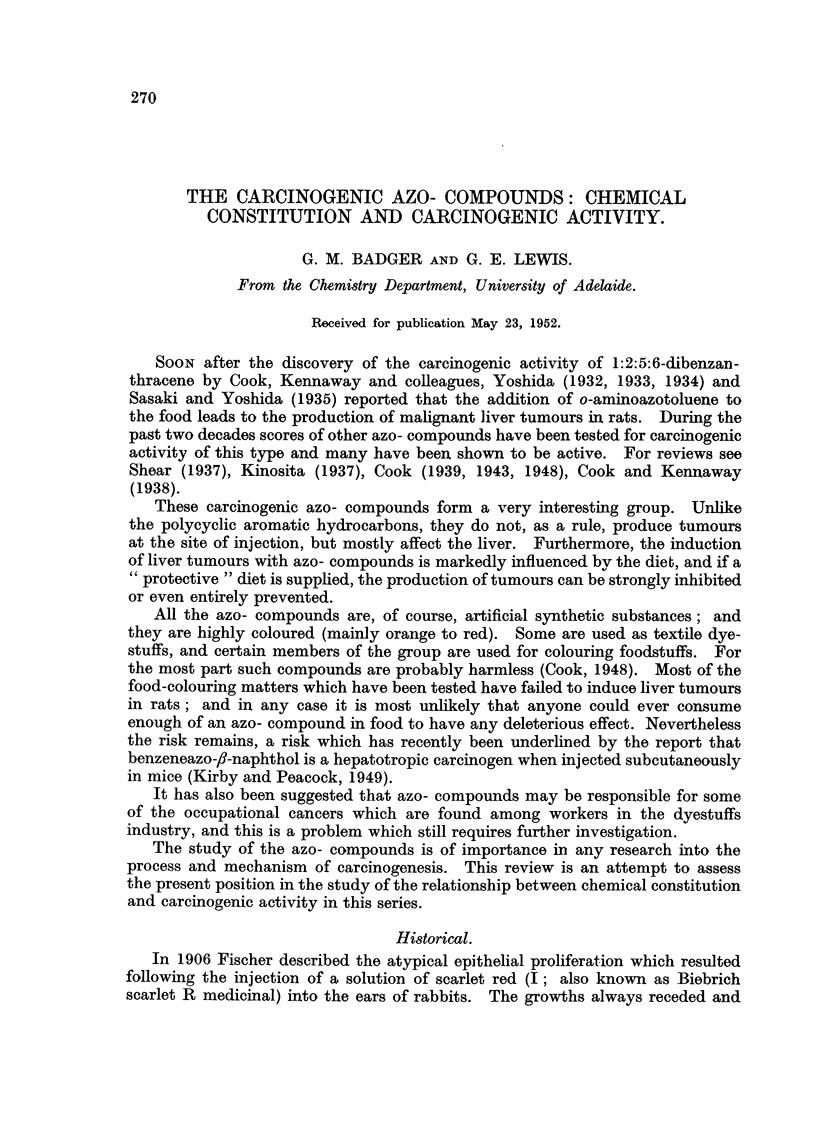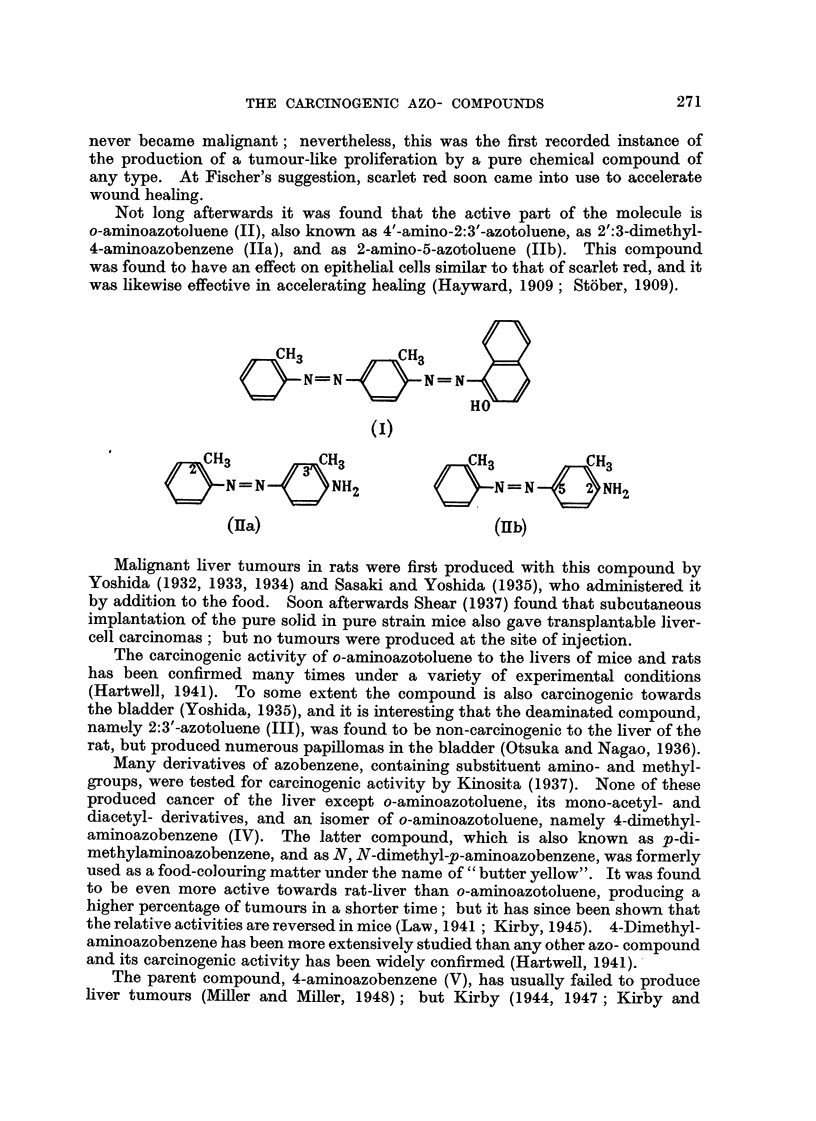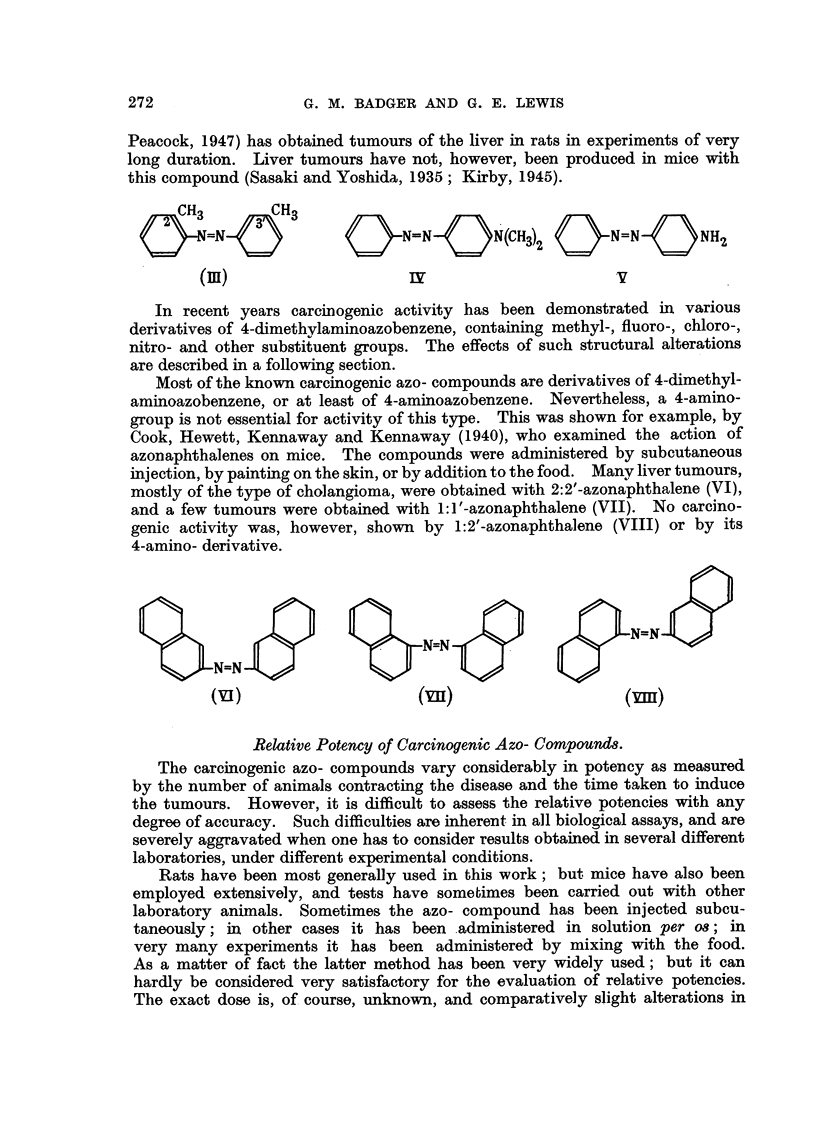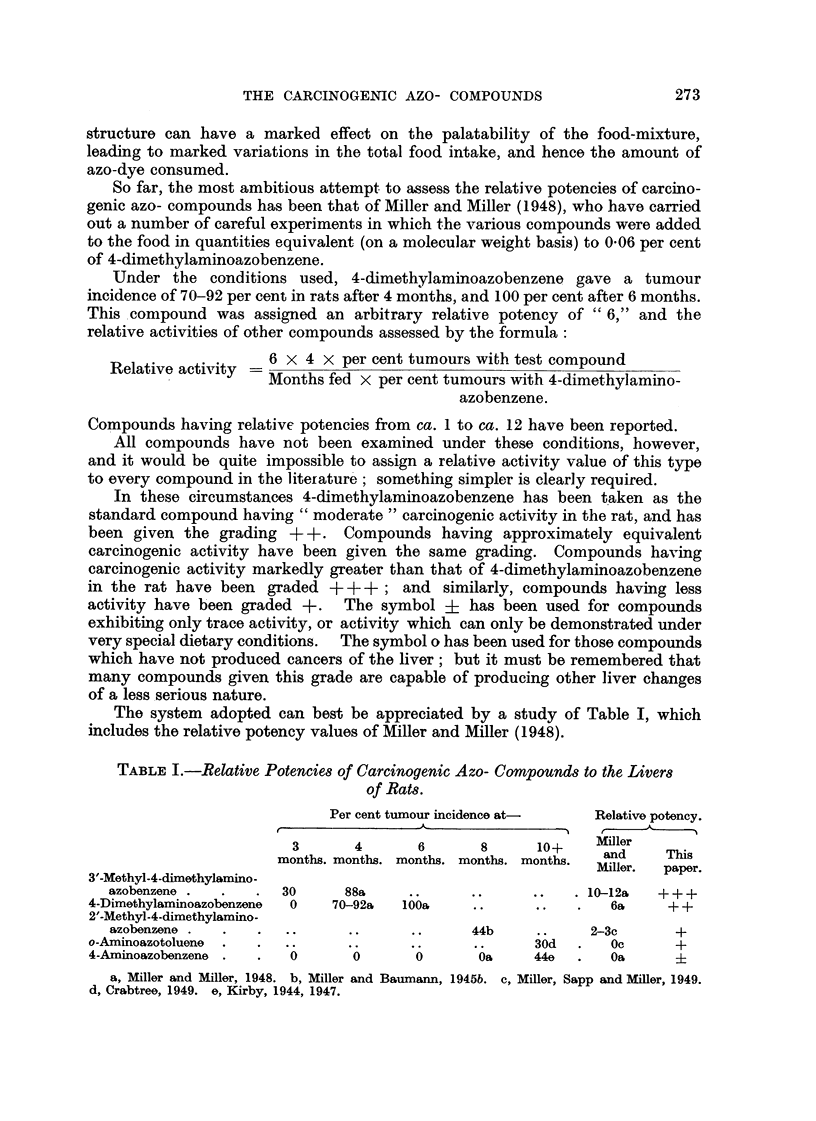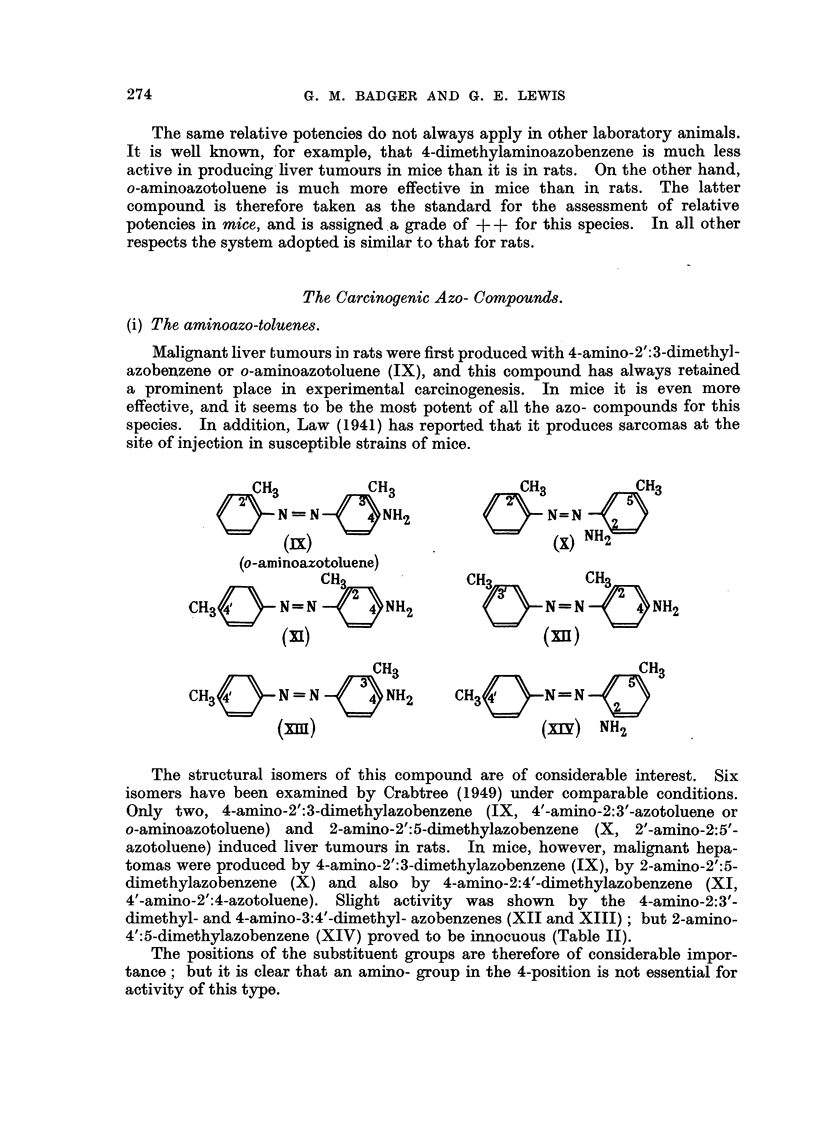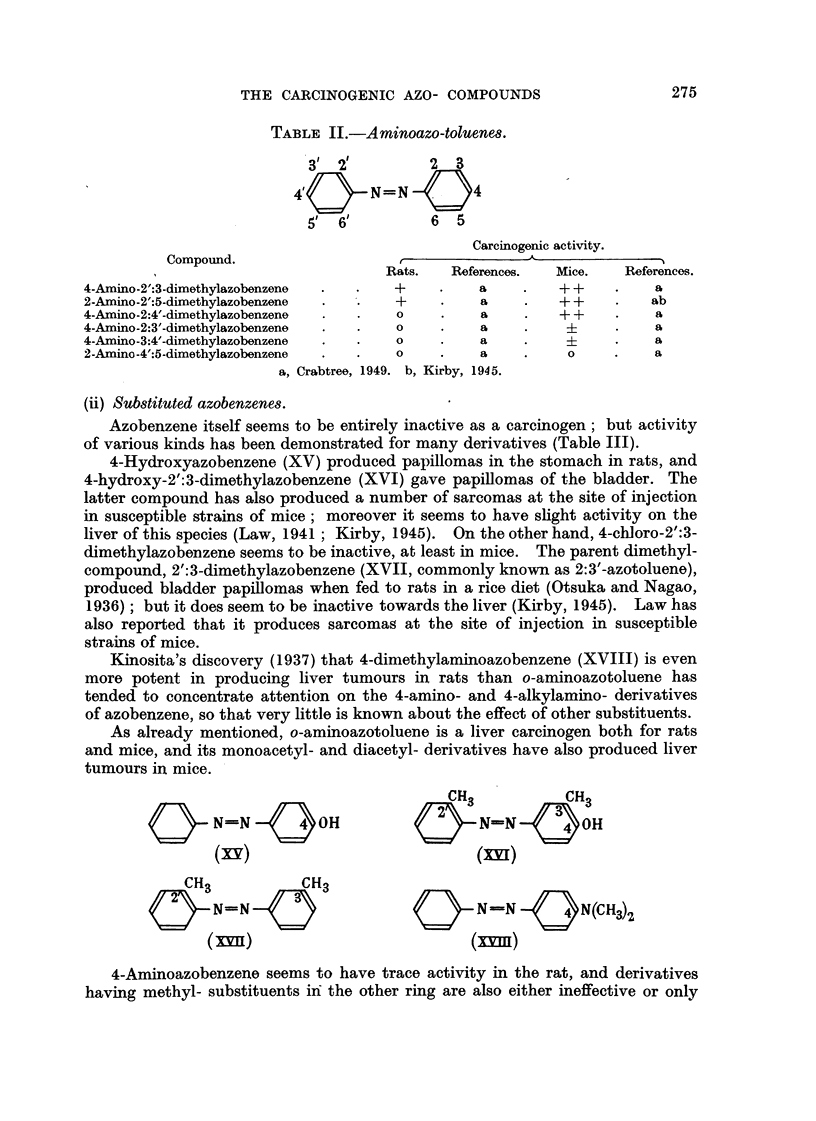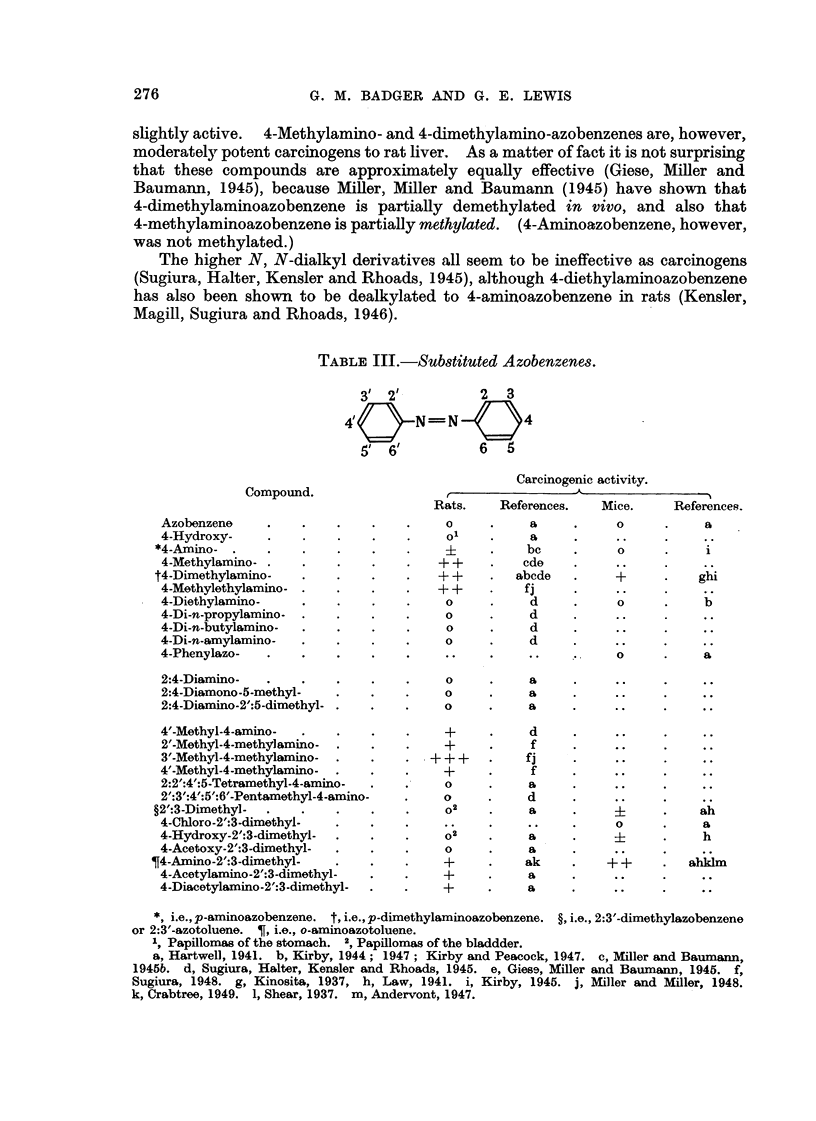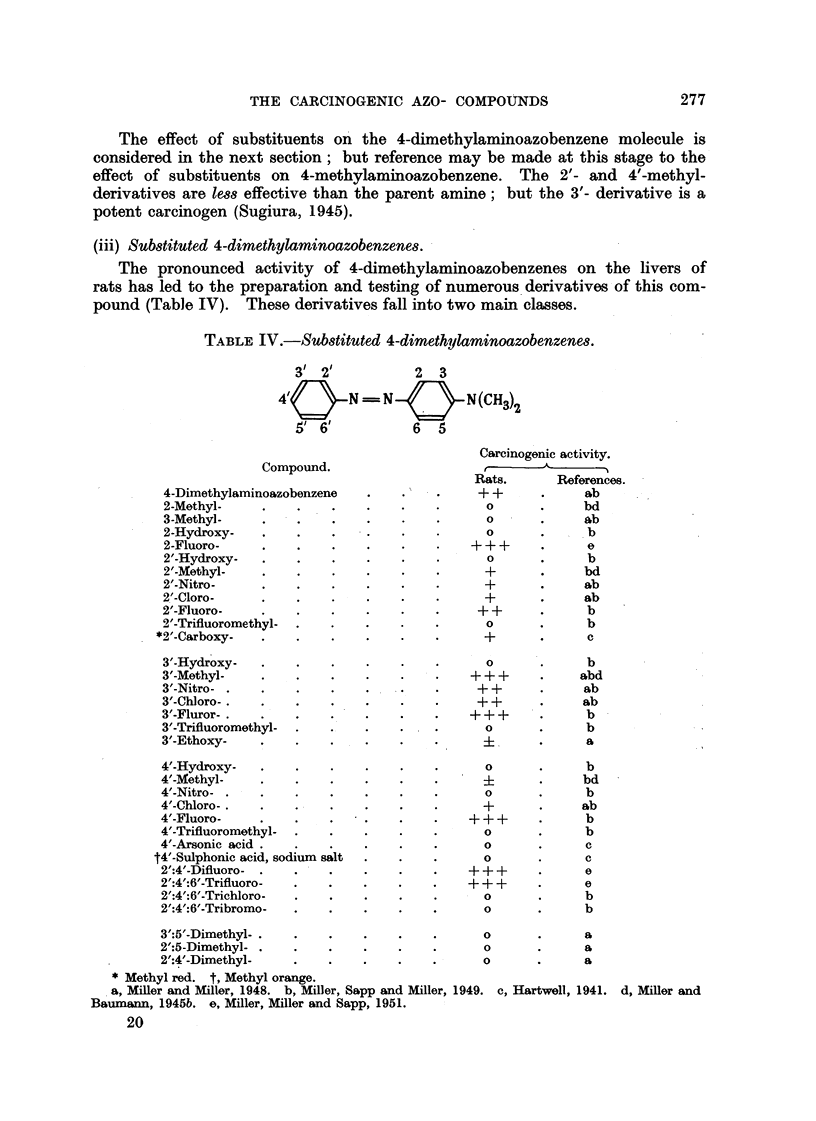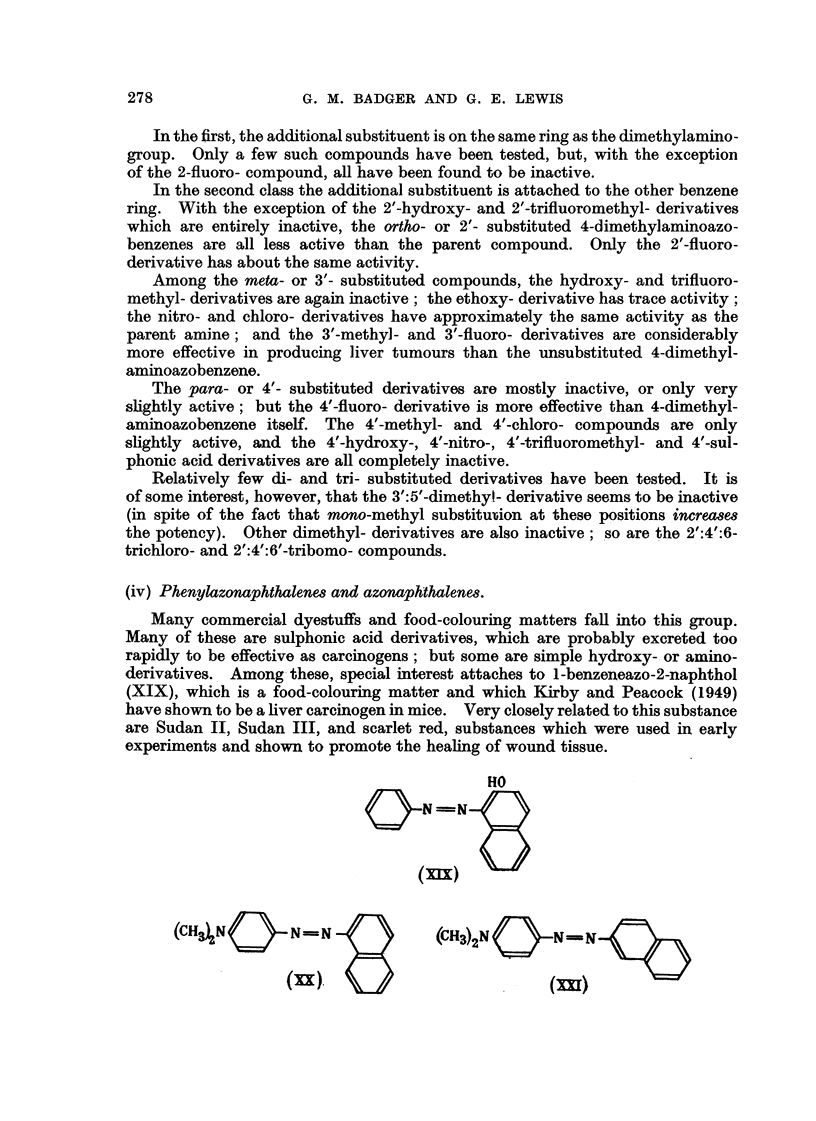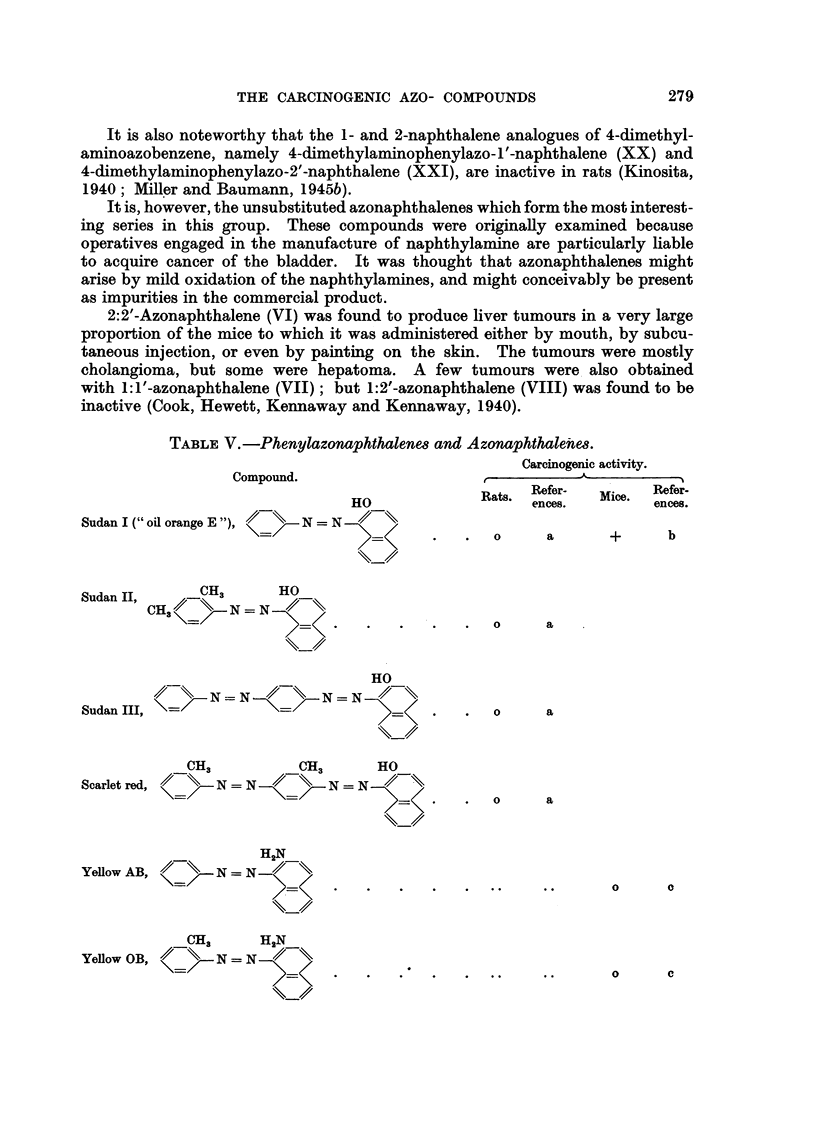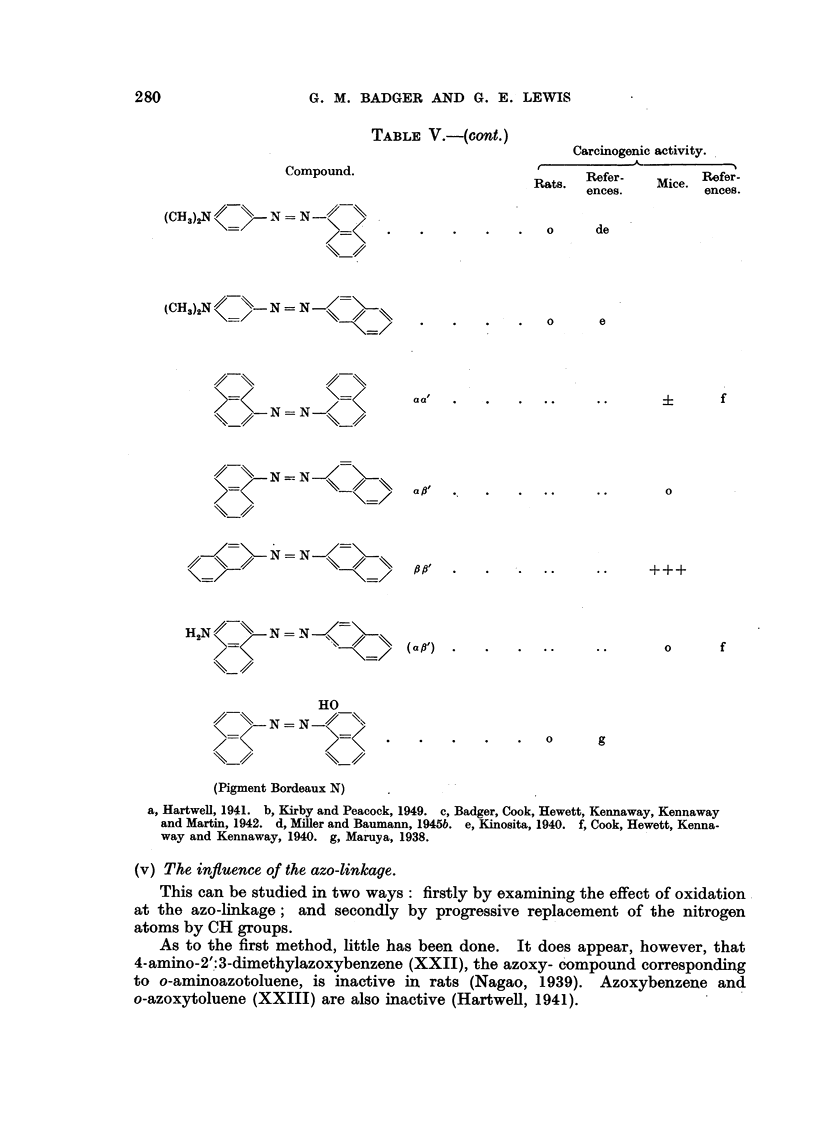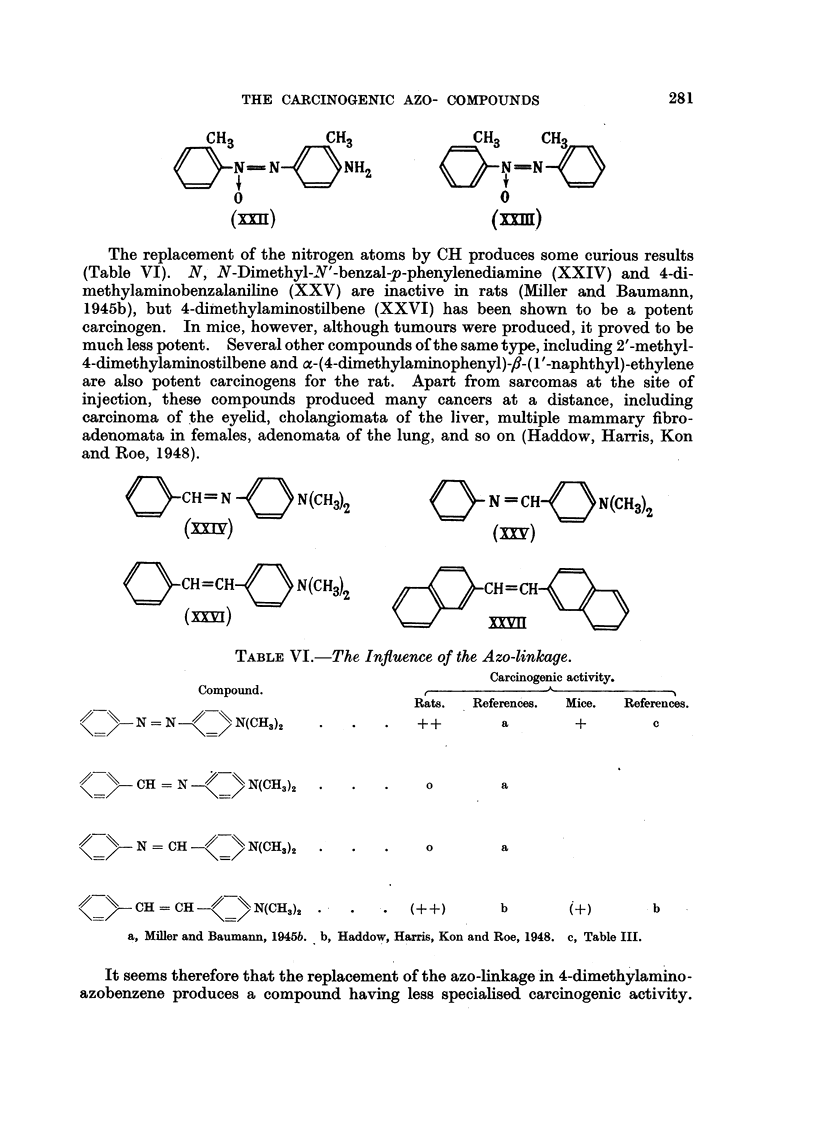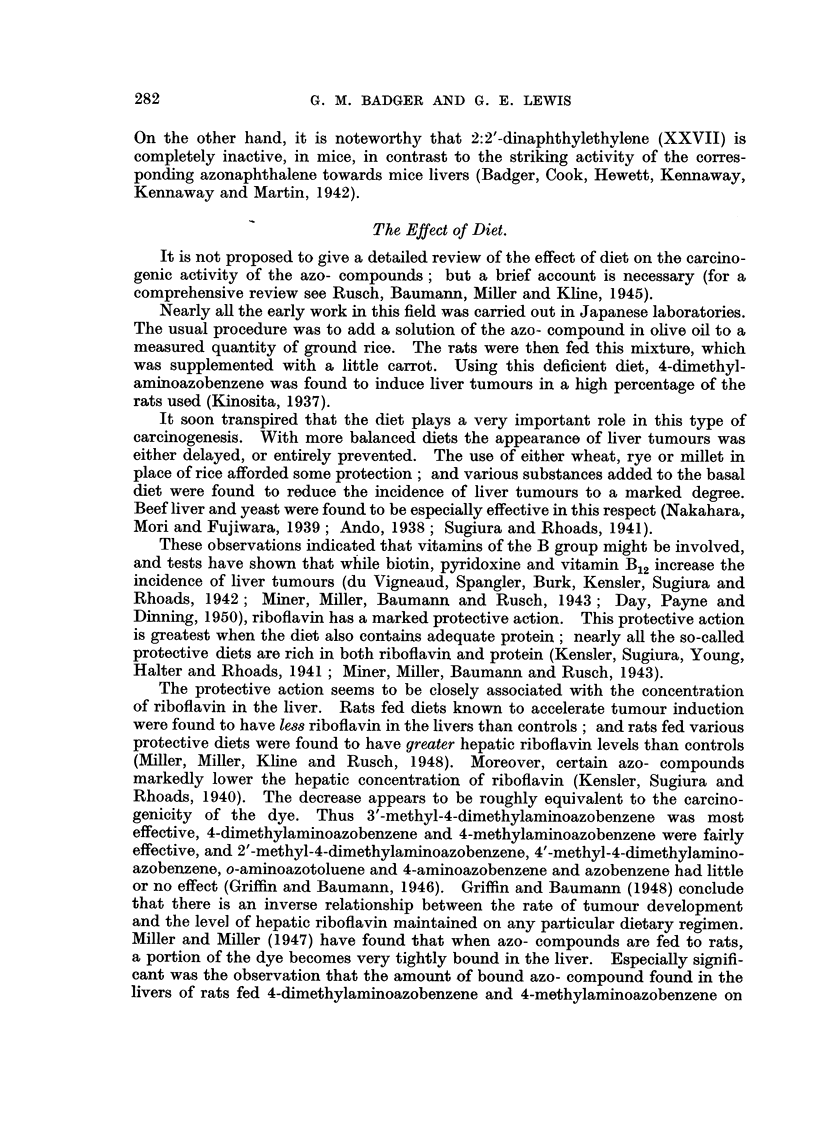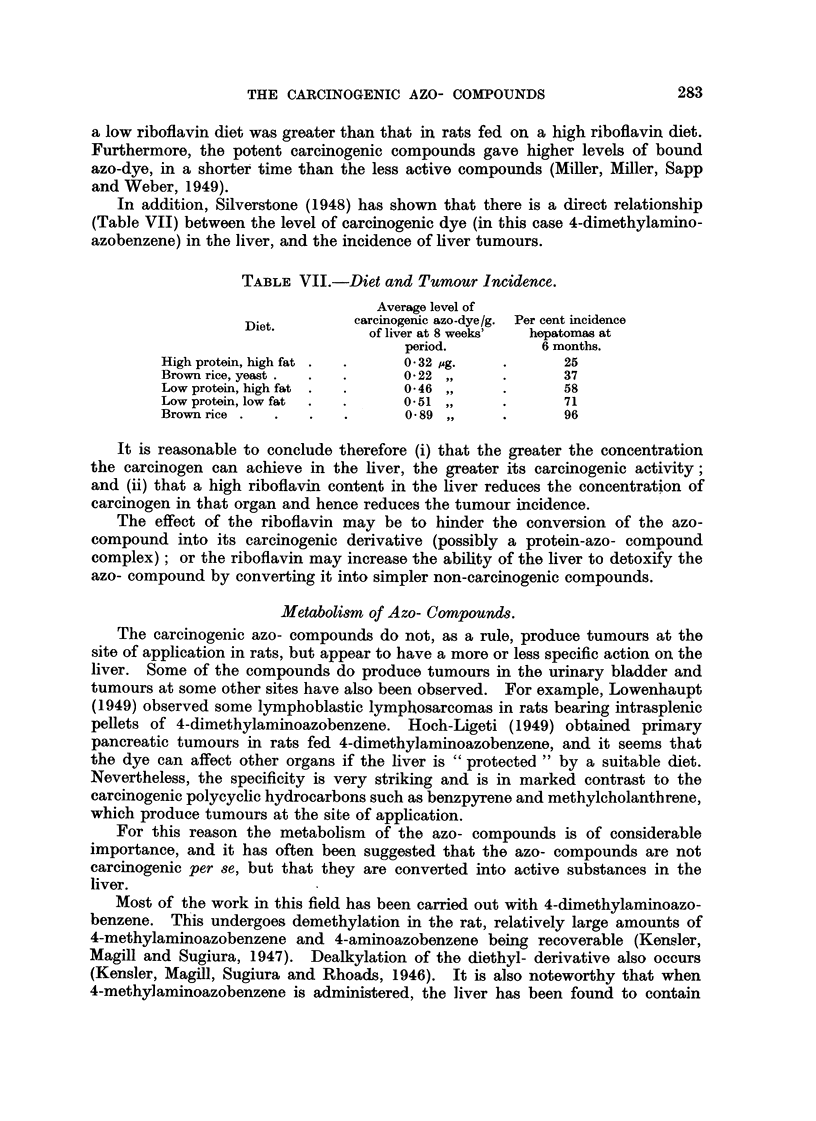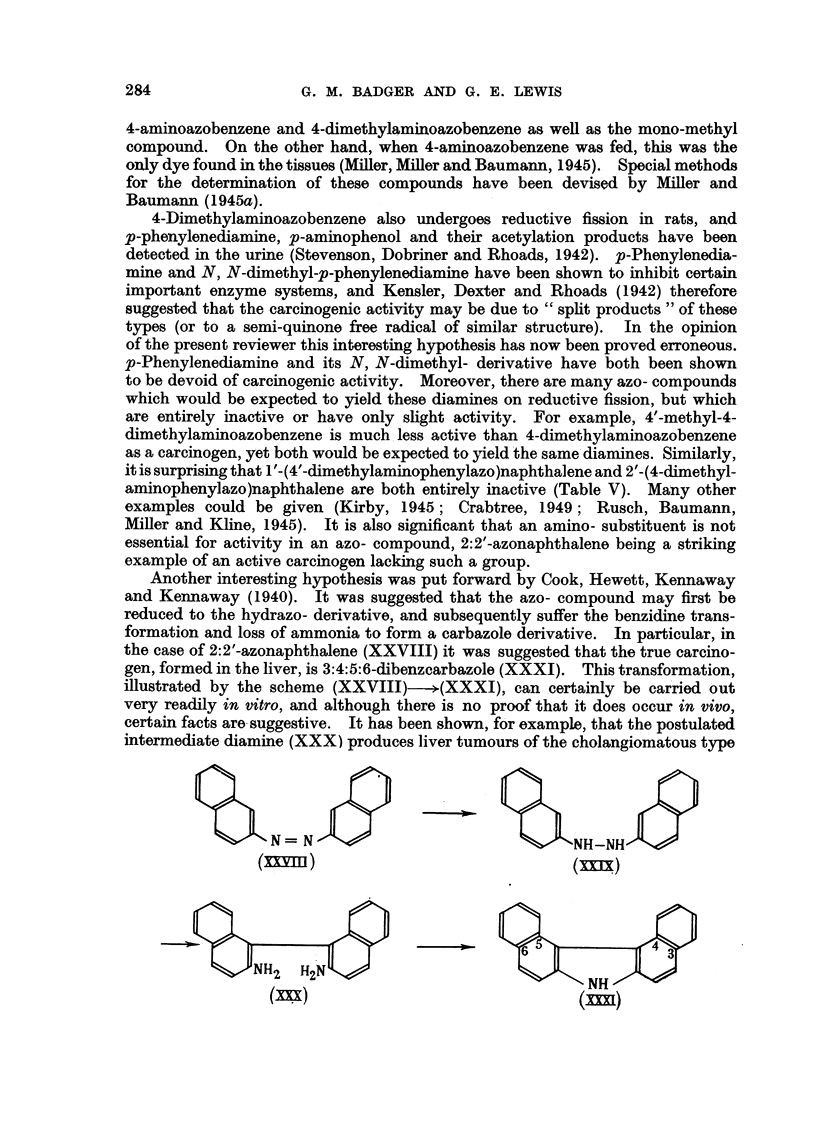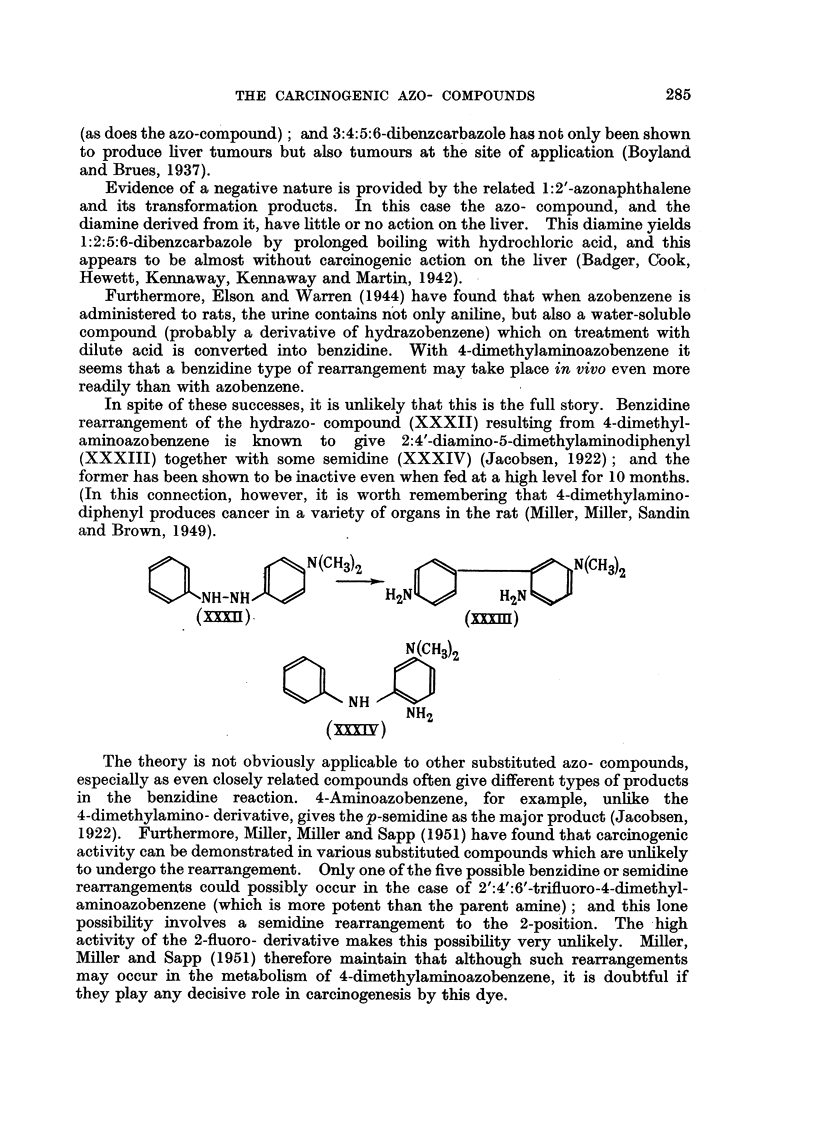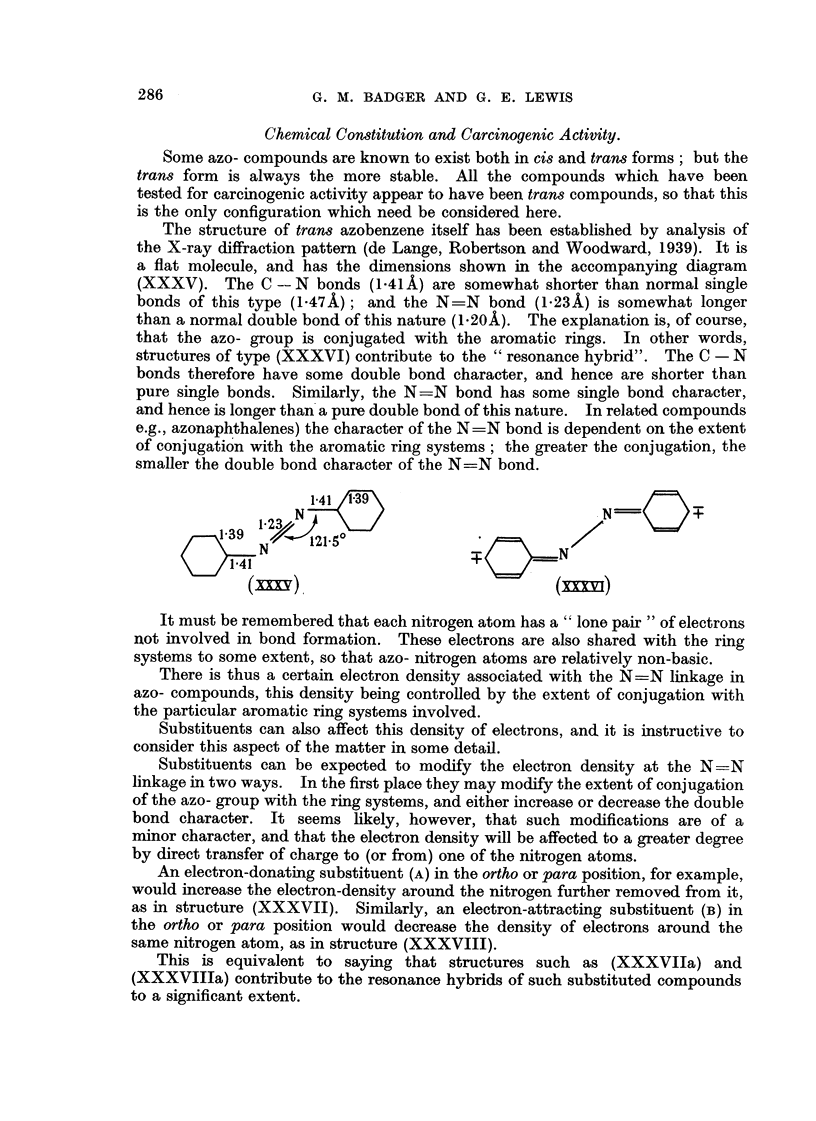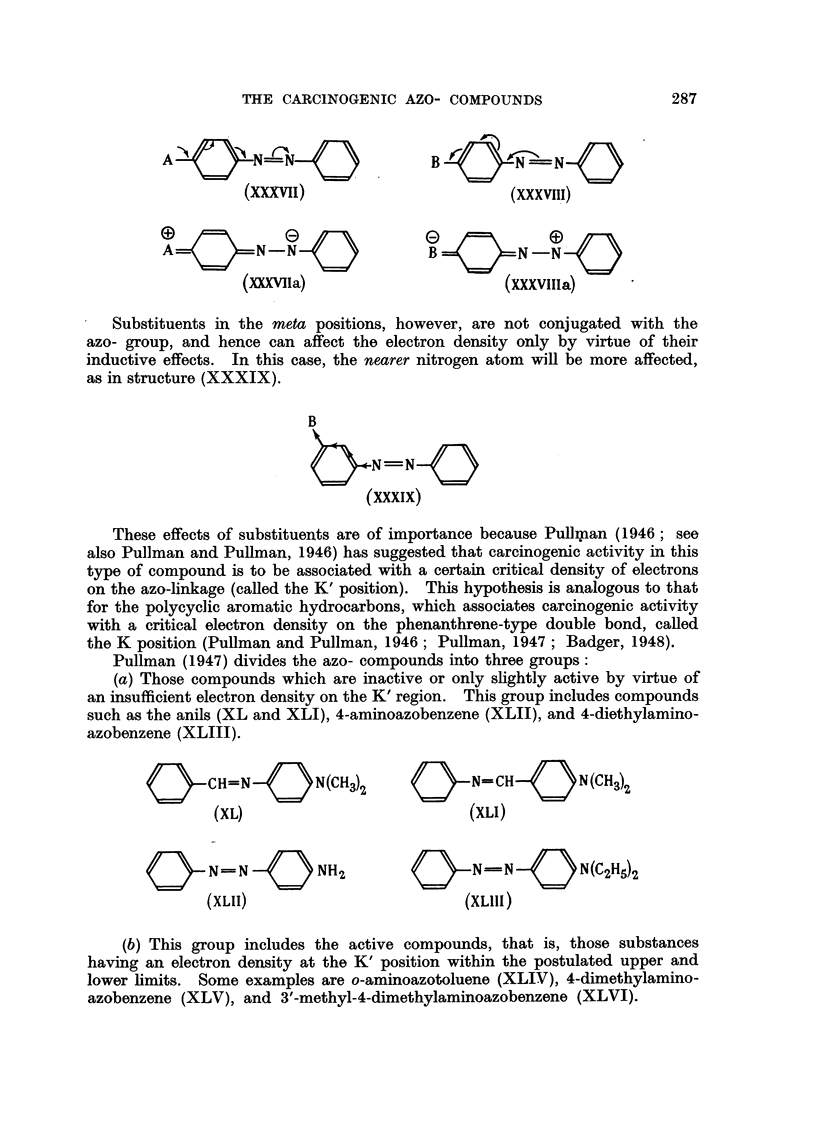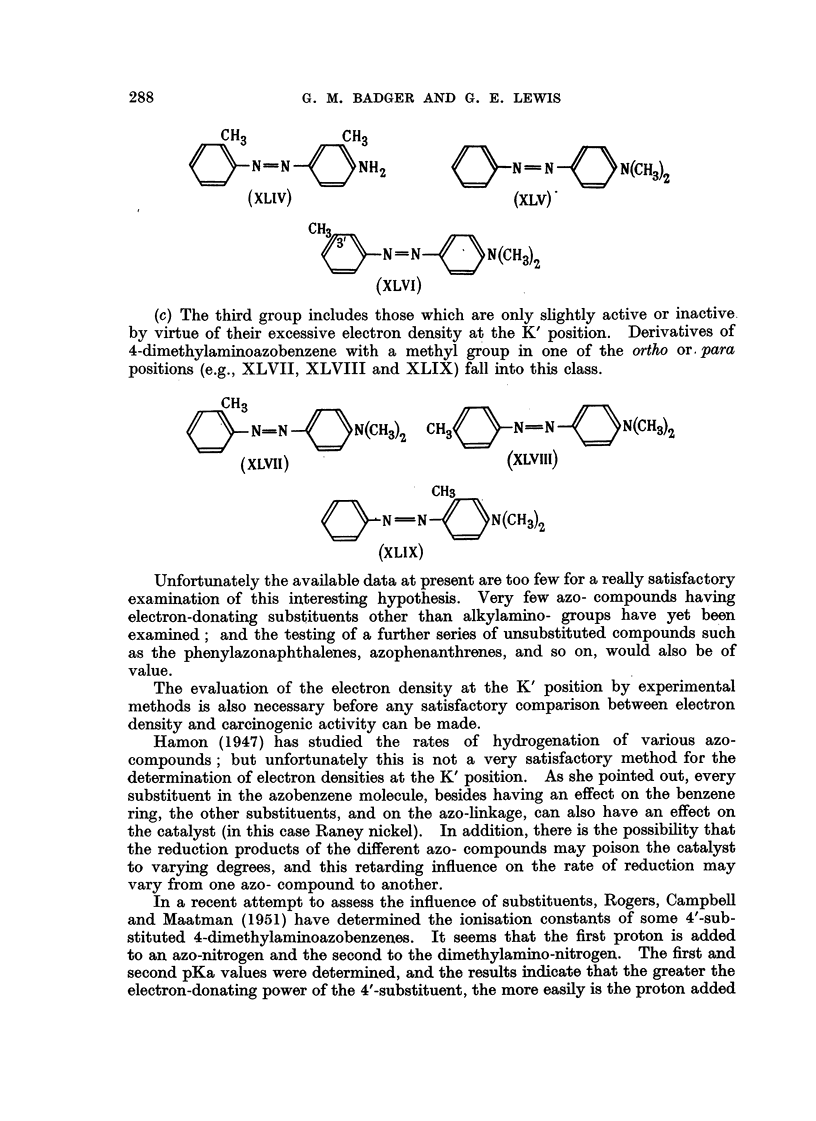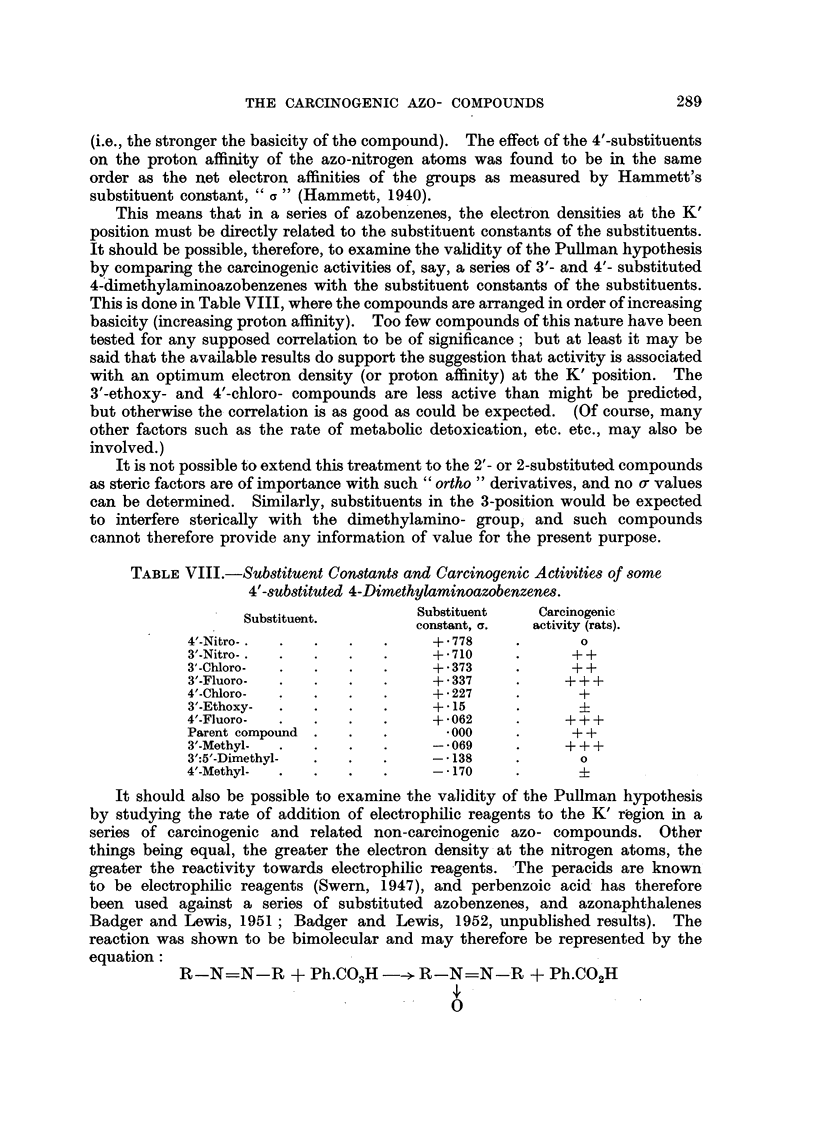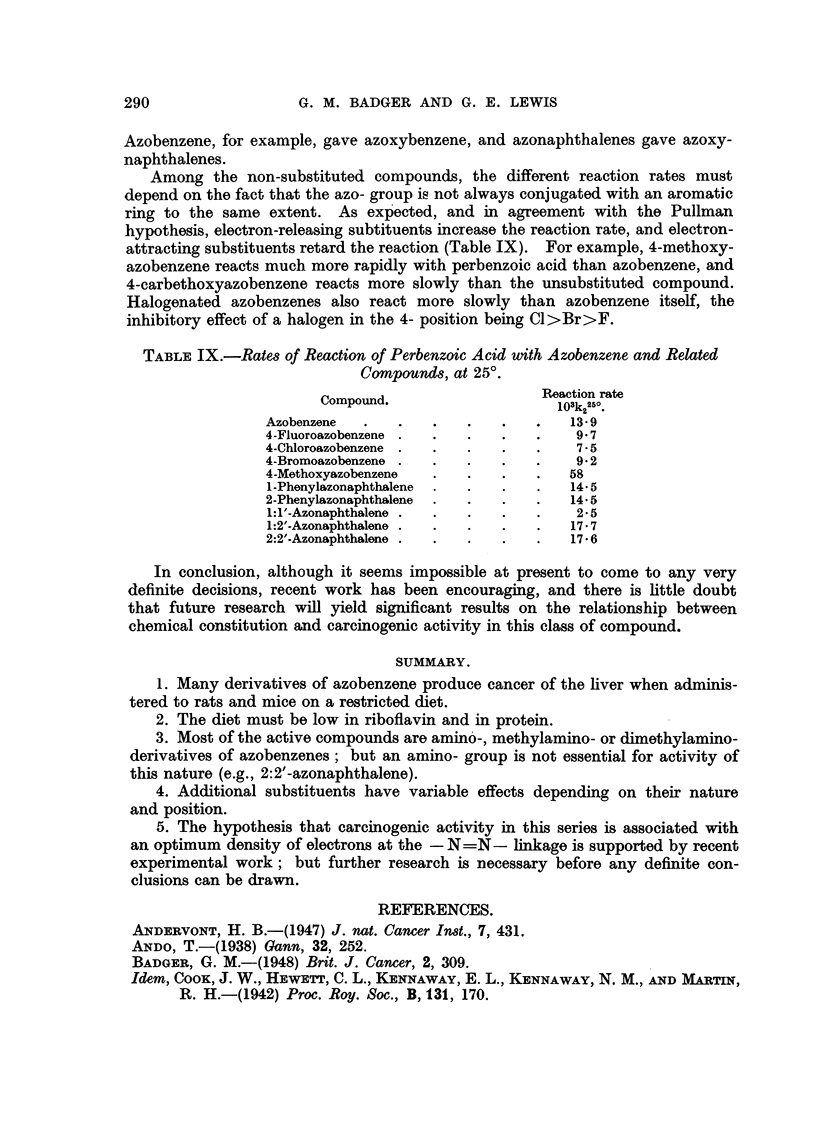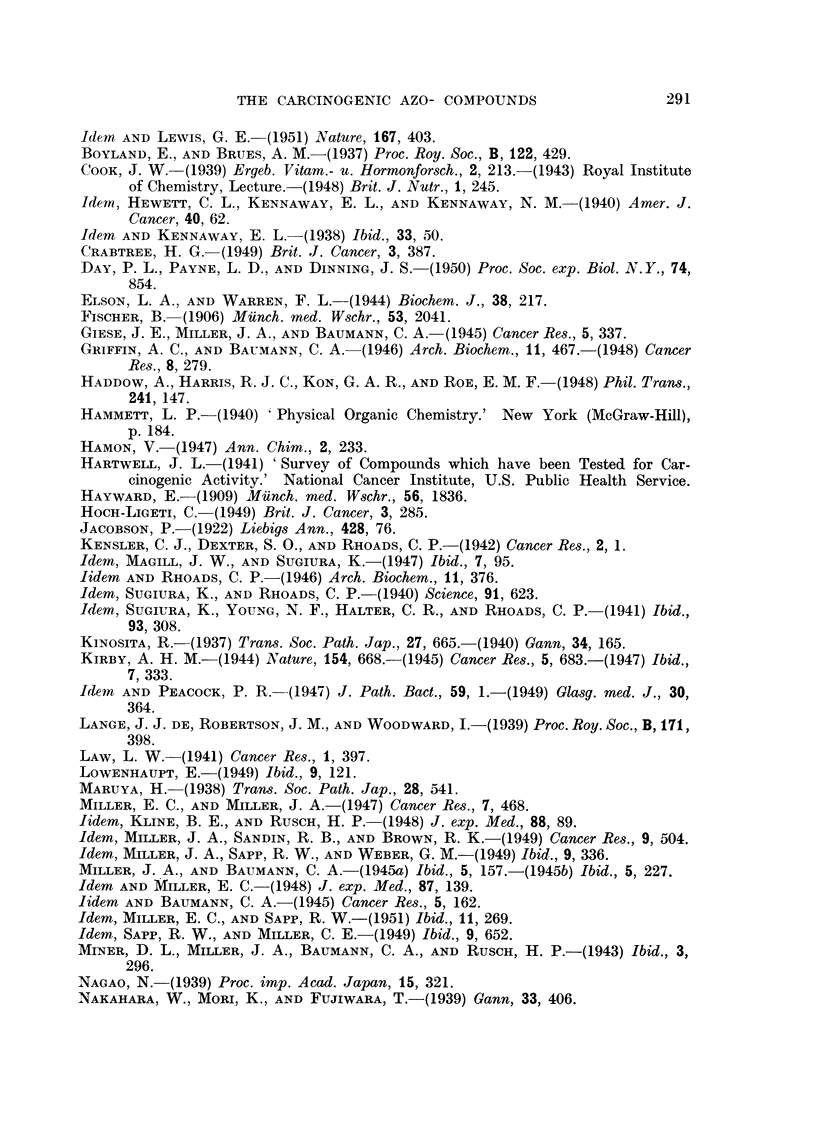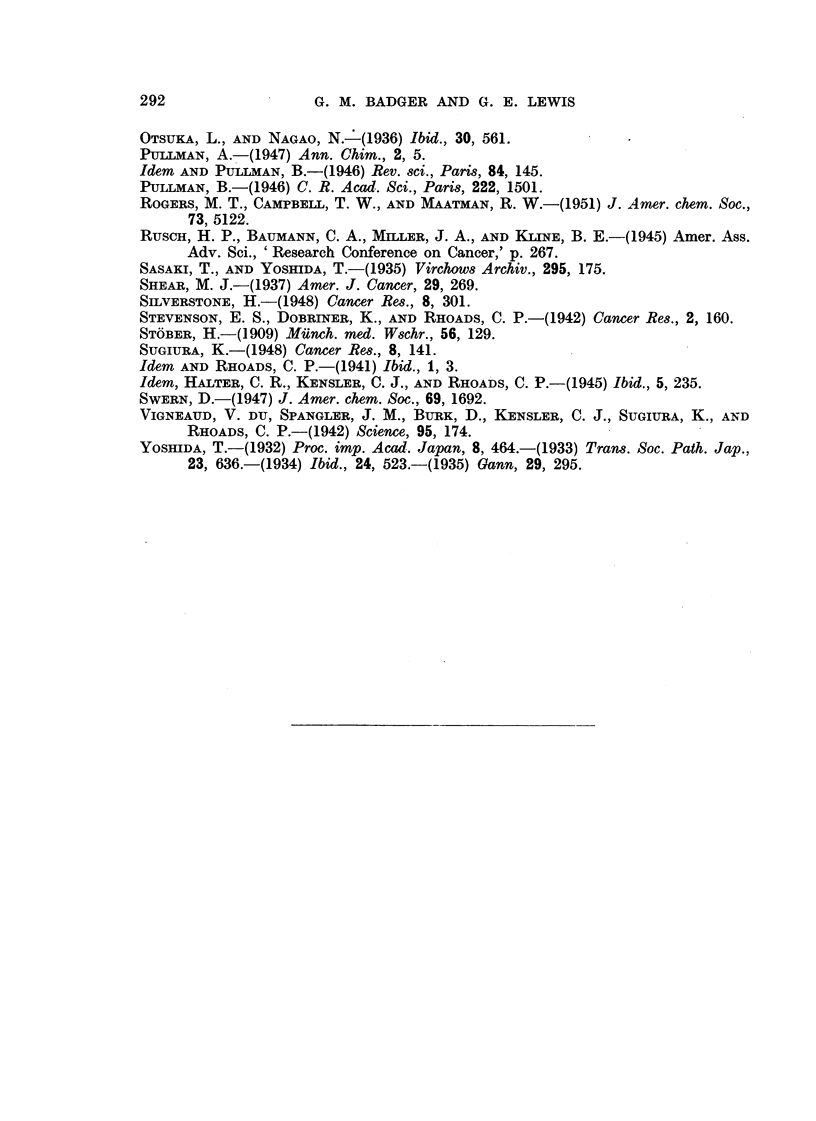# The Carcinogenic Azo- Compounds: Chemical Constitution and Carcinogenic Activity

**DOI:** 10.1038/bjc.1952.32

**Published:** 1952-09

**Authors:** G. M. Badger, G. E. Lewis


### THE CARCINOGENIC AZO- COMPOUNDS: CHEMICAL CONSTITUTION AND CARCINOGENIC ACTIVITY.

G. M. BADGER AND G. E. LEWIS.

*From the Chemistry Department, University of Adelaide.*

Received for publication May 23, 1952.

SOON after the discovery of the carcinogenic activity of 1:2:5:6-dibenzanthracene by Cook, Kennaway and colleagues, Yoshida (1932, 1933, 1934) and Sasaki and Yoshida (1935) reported that the addition of *o*-aminoazotoluene to the food leads to the production of malignant liver tumours in rats. During the past two decades scores of other azo- compounds have been tested for carcinogenic activity of this type and many have been shown to be active. For reviews see Shear (1937), Kinosita (1937), Cook (1939, 1943, 1948), Cook and Kennaway (1938).

These carcinogenic azo- compounds form a very interesting group. Unlike the polycyclic aromatic hydrocarbons, they do not, as a rule, produce tumours at the site of injection, but mostly affect the liver. Furthermore, the induction of liver tumours with azo- compounds is markedly influenced by the diet, and if a "protective" diet is supplied, the production of tumours can be strongly inhibited or even entirely prevented.

All the azo- compounds are, of course, artificial synthetic substances; and they are highly coloured (mainly orange to red). Some are used as textile dyestuffs, and certain members of the group are used for colouring foodstuffs. For the most part such compounds are probably harmless (Cook, 1948). Most of the food-colouring matters which have been tested have failed to induce liver tumours in rats; and in any case it is most unlikely that anyone could ever consume enough of an azo- compound in food to have any deleterious effect. Nevertheless the risk remains, a risk which has recently been underlined by the report that benzeneazo-$\beta$-naphthol is a hepatotropic carcinogen when injected subcutaneously in mice (Kirby and Peacock, 1949).

It has also been suggested that azo- compounds may be responsible for some of the occupational cancers which are found among workers in the dyestuffs industry, and this is a problem which still requires further investigation.

The study of the azo- compounds is of importance in any research into the process and mechanism of carcinogenesis. This review is an attempt to assess the present position in the study of the relationship between chemical constitution and carcinogenic activity in this series.

### *Historical.*

In 1906 Fischer described the atypical epithelial proliferation which resulted following the injection of a solution of scarlet red (I; also known as Biebrich scarlet R medicinal) into the ears of rabbits. The growths always receded and never became malignant; nevertheless, this was the first recorded instance of the production of a tumour-like proliferation by a pure chemical compound of any type. At Fischer's suggestion, scarlet red soon came into use to accelerate wound healing.

Not long afterwards it was found that the active part of the molecule is *o*-aminoazotoluene (II), also known as 4′-amino-2:3′-azotoluene, as 2′:3-dimethyl-4-aminoazobenzene (IIa), and as 2-amino-5-azotoluene (IIb). This compound was found to have an effect on epithelial cells similar to that of scarlet red, and it was likewise effective in accelerating healing (Hayward, 1909; Stöber, 1909).

(I)

(IIa)

(IIb)

Malignant liver tumours in rats were first produced with this compound by Yoshida (1932, 1933, 1934) and Sasaki and Yoshida (1935), who administered it by addition to the food. Soon afterwards Shear (1937) found that subcutaneous implantation of the pure solid in pure strain mice also gave transplantable liver-cell carcinomas; but no tumours were produced at the site of injection.

The carcinogenic activity of *o*-aminoazotoluene to the livers of mice and rats has been confirmed many times under a variety of experimental conditions (Hartwell, 1941). To some extent the compound is also carcinogenic towards the bladder (Yoshida, 1935), and it is interesting that the deaminated compound, namely 2:3′-azotoluene (III), was found to be non-carcinogenic to the liver of the rat, but produced numerous papillomas in the bladder (Otsuka and Nagao, 1936).

Many derivatives of azobenzene, containing substituent amino- and methyl-groups, were tested for carcinogenic activity by Kinosita (1937). None of these produced cancer of the liver except *o*-aminoazotoluene, its mono-acetyl- and diacetyl- derivatives, and an isomer of *o*-aminoazotoluene, namely 4-dimethylaminoazobenzene (IV). The latter compound, which is also known as *p*-dimethylaminoazobenzene, and as *N*, *N*-dimethyl-*p*-aminoazobenzene, was formerly used as a food-colouring matter under the name of "butter yellow". It was found to be even more active towards rat-liver than *o*-aminoazotoluene, producing a higher percentage of tumours in a shorter time; but it has since been shown that the relative activities are reversed in mice (Law, 1941; Kirby, 1945). 4-Dimethylaminoazobenzene has been more extensively studied than any other azo- compound and its carcinogenic activity has been widely confirmed (Hartwell, 1941).

The parent compound, 4-aminoazobenzene (V), has usually failed to produce liver tumours (Miller and Miller, 1948); but Kirby (1944, 1947; Kirby and

Peacock, 1947) has obtained tumours of the liver in rats in experiments of very long duration. Liver tumours have not, however, been produced in mice with this compound (Sasaki and Yoshida, 1935 ; Kirby, 1945).

(III)

IV

V

In recent years carcinogenic activity has been demonstrated in various derivatives of 4-dimethylaminoazobenzene, containing methyl-, fluoro-, chloro-, nitro- and other substituent groups. The effects of such structural alterations are described in a following section.

Most of the known carcinogenic azo- compounds are derivatives of 4-dimethylaminoazobenzene, or at least of 4-aminoazobenzene. Nevertheless, a 4-amino-group is not essential for activity of this type. This was shown for example, by Cook, Hewett, Kennaway and Kennaway (1940), who examined the action of azonaphthalenes on mice. The compounds were administered by subcutaneous injection, by painting on the skin, or by addition to the food. Many liver tumours, mostly of the type of cholangioma, were obtained with 2:2′-azonaphthalene (VI), and a few tumours were obtained with 1:1′-azonaphthalene (VII). No carcinogenic activity was, however, shown by 1:2′-azonaphthalene (VIII) or by its 4-amino- derivative.

(VI)

(VII)

(VIII)

*Relative Potency of Carcinogenic Azo- Compounds.*

The carcinogenic azo- compounds vary considerably in potency as measured by the number of animals contracting the disease and the time taken to induce the tumours. However, it is difficult to assess the relative potencies with any degree of accuracy. Such difficulties are inherent in all biological assays, and are severely aggravated when one has to consider results obtained in several different laboratories, under different experimental conditions.

Rats have been most generally used in this work ; but mice have also been employed extensively, and tests have sometimes been carried out with other laboratory animals. Sometimes the azo- compound has been injected subcutaneously ; in other cases it has been administered in solution *per os* ; in very many experiments it has been administered by mixing with the food. As a matter of fact the latter method has been very widely used ; but it can hardly be considered very satisfactory for the evaluation of relative potencies. The exact dose is, of course, unknown, and comparatively slight alterations in structure can have a marked effect on the palatability of the food-mixture, leading to marked variations in the total food intake, and hence the amount of azo-dye consumed.

So far, the most ambitious attempt to assess the relative potencies of carcinogenic azo- compounds has been that of Miller and Miller (1948), who have carried out a number of careful experiments in which the various compounds were added to the food in quantities equivalent (on a molecular weight basis) to 0·06 per cent of 4-dimethylaminoazobenzene.

Under the conditions used, 4-dimethylaminoazobenzene gave a tumour incidence of 70–92 per cent in rats after 4 months, and 100 per cent after 6 months. This compound was assigned an arbitrary relative potency of " 6," and the relative activities of other compounds assessed by the formula :

$$\text{Relative activity} = \frac{6 \times 4 \times \text{per cent tumours with test compound}}{\text{Months fed} \times \text{per cent tumours with 4-dimethylaminoazobenzene.}}$$

Compounds having relative potencies from *ca.* 1 to *ca.* 12 have been reported.

All compounds have not been examined under these conditions, however, and it would be quite impossible to assign a relative activity value of this type to every compound in the literature ; something simpler is clearly required.

In these circumstances 4-dimethylaminoazobenzene has been taken as the standard compound having " moderate " carcinogenic activity in the rat, and has been given the grading ++. Compounds having approximately equivalent carcinogenic activity have been given the same grading. Compounds having carcinogenic activity markedly greater than that of 4-dimethylaminoazobenzene in the rat have been graded +++ ; and similarly, compounds having less activity have been graded +. The symbol ± has been used for compounds exhibiting only trace activity, or activity which can only be demonstrated under very special dietary conditions. The symbol o has been used for those compounds which have not produced cancers of the liver ; but it must be remembered that many compounds given this grade are capable of producing other liver changes of a less serious nature.

The system adopted can best be appreciated by a study of Table I, which includes the relative potency values of Miller and Miller (1948).

TABLE I.—*Relative Potencies of Carcinogenic Azo- Compounds to the Livers of Rats.*

| | Per cent tumour incidence at— | | | | | Relative potency. | |
|---|---|---|---|---|---|---|---|
| | 3 months. | 4 months. | 6 months. | 8 months. | 10+ months. | Miller and Miller. | This paper. |
| 3′-Methyl-4-dimethylamino-azobenzene . . . | 30 | 88a | .. | .. | .. | . 10–12a | +++ |
| 4-Dimethylaminoazobenzene | 0 | 70–92a | 100a | .. | .. | . 6a | ++ |
| 2′-Methyl-4-dimethylamino-azobenzene . . . | .. | .. | .. | 44b | .. | 2–3c | + |
| *o*-Aminoazotoluene . . | .. | .. | .. | .. | 30d | . 0c | + |
| 4-Aminoazobenzene . . | 0 | 0 | 0 | 0a | 44e | . 0a | ± |

a, Miller and Miller, 1948. b, Miller and Baumann, 1945*b*. c, Miller, Sapp and Miller, 1949. d, Crabtree, 1949. e, Kirby, 1944, 1947.

The same relative potencies do not always apply in other laboratory animals. It is well known, for example, that 4-dimethylaminoazobenzene is much less active in producing liver tumours in mice than it is in rats. On the other hand, *o*-aminoazotoluene is much more effective in mice than in rats. The latter compound is therefore taken as the standard for the assessment of relative potencies in *mice*, and is assigned a grade of ++ for this species. In all other respects the system adopted is similar to that for rats.

### *The Carcinogenic Azo- Compounds.*

(i) *The aminoazo-toluenes.*

Malignant liver tumours in rats were first produced with 4-amino-2′:3-dimethylazobenzene or *o*-aminoazotoluene (IX), and this compound has always retained a prominent place in experimental carcinogenesis. In mice it is even more effective, and it seems to be the most potent of all the azo- compounds for this species. In addition, Law (1941) has reported that it produces sarcomas at the site of injection in susceptible strains of mice.

(IX)
(*o*-aminoazotoluene)

(X)

(XI)

(XII)

(XIII)

(XIV)

The structural isomers of this compound are of considerable interest. Six isomers have been examined by Crabtree (1949) under comparable conditions. Only two, 4-amino-2′:3-dimethylazobenzene (IX, 4′-amino-2:3′-azotoluene or *o*-aminoazotoluene) and 2-amino-2′:5-dimethylazobenzene (X, 2′-amino-2:5′-azotoluene) induced liver tumours in rats. In mice, however, malignant hepatomas were produced by 4-amino-2′:3-dimethylazobenzene (IX), by 2-amino-2′:5-dimethylazobenzene (X) and also by 4-amino-2:4′-dimethylazobenzene (XI, 4′-amino-2′:4-azotoluene). Slight activity was shown by the 4-amino-2:3′-dimethyl- and 4-amino-3:4′-dimethyl- azobenzenes (XII and XIII); but 2-amino-4′:5-dimethylazobenzene (XIV) proved to be innocuous (Table II).

The positions of the substituent groups are therefore of considerable importance; but it is clear that an amino- group in the 4-position is not essential for activity of this type.

TABLE II.—*Aminoazo-toluenes.*

| Compound. | Carcinogenic activity. Rats. | References. | Mice. | References. |
|---|---|---|---|---|
| 4-Amino-2′:3-dimethylazobenzene | + | a | ++ | a |
| 2-Amino-2′:5-dimethylazobenzene | + | a | ++ | ab |
| 4-Amino-2:4′-dimethylazobenzene | o | a | ++ | a |
| 4-Amino-2:3′-dimethylazobenzene | o | a | ± | a |
| 4-Amino-3:4′-dimethylazobenzene | o | a | ± | a |
| 2-Amino-4′:5-dimethylazobenzene | o | a | o | a |

a, Crabtree, 1949. b, Kirby, 1945.

(ii) *Substituted azobenzenes.*

Azobenzene itself seems to be entirely inactive as a carcinogen ; but activity of various kinds has been demonstrated for many derivatives (Table III).

4-Hydroxyazobenzene (XV) produced papillomas in the stomach in rats, and 4-hydroxy-2′:3-dimethylazobenzene (XVI) gave papillomas of the bladder. The latter compound has also produced a number of sarcomas at the site of injection in susceptible strains of mice ; moreover it seems to have slight activity on the liver of this species (Law, 1941 ; Kirby, 1945). On the other hand, 4-chloro-2′:3-dimethylazobenzene seems to be inactive, at least in mice. The parent dimethyl-compound, 2′:3-dimethylazobenzene (XVII, commonly known as 2:3′-azotoluene), produced bladder papillomas when fed to rats in a rice diet (Otsuka and Nagao, 1936) ; but it does seem to be inactive towards the liver (Kirby, 1945). Law has also reported that it produces sarcomas at the site of injection in susceptible strains of mice.

Kinosita's discovery (1937) that 4-dimethylaminoazobenzene (XVIII) is even more potent in producing liver tumours in rats than *o*-aminoazotoluene has tended to concentrate attention on the 4-amino- and 4-alkylamino- derivatives of azobenzene, so that very little is known about the effect of other substituents.

As already mentioned, *o*-aminoazotoluene is a liver carcinogen both for rats and mice, and its monoacetyl- and diacetyl- derivatives have also produced liver tumours in mice.

(XV)

(XVI)

(XVII)

(XVIII)

4-Aminoazobenzene seems to have trace activity in the rat, and derivatives having methyl- substituents in the other ring are also either ineffective or only slightly active. 4-Methylamino- and 4-dimethylamino-azobenzenes are, however, moderately potent carcinogens to rat liver. As a matter of fact it is not surprising that these compounds are approximately equally effective (Giese, Miller and Baumann, 1945), because Miller, Miller and Baumann (1945) have shown that 4-dimethylaminoazobenzene is partially demethylated *in vivo*, and also that 4-methylaminoazobenzene is partially *methylated*. (4-Aminoazobenzene, however, was not methylated.)

The higher *N*, *N*-dialkyl derivatives all seem to be ineffective as carcinogens (Sugiura, Halter, Kensler and Rhoads, 1945), although 4-diethylaminoazobenzene has also been shown to be dealkylated to 4-aminoazobenzene in rats (Kensler, Magill, Sugiura and Rhoads, 1946).

TABLE III.—*Substituted Azobenzenes.*

| Compound. | Carcinogenic activity. Rats. | References. | Mice. | References. |
|---|---|---|---|---|
| Azobenzene | o | a | o | a |
| 4-Hydroxy- | o[1] | a | .. | .. |
| *4-Amino- | ± | bc | o | i |
| 4-Methylamino- | ++ | cde | .. | .. |
| †4-Dimethylamino- | ++ | abcde | + | ghi |
| 4-Methylethylamino- | ++ | fj | .. | .. |
| 4-Diethylamino- | o | d | o | b |
| 4-Di-*n*-propylamino- | o | d | .. | .. |
| 4-Di-*n*-butylamino- | o | d | .. | .. |
| 4-Di-*n*-amylamino- | o | d | .. | .. |
| 4-Phenylazo- | .. | .. | o | a |
| 2:4-Diamino- | o | a | .. | .. |
| 2:4-Diamono-5-methyl- | o | a | .. | .. |
| 2:4-Diamino-2′:5-dimethyl- | o | a | .. | .. |
| 4′-Methyl-4-amino- | + | d | .. | .. |
| 2′-Methyl-4-methylamino- | + | f | .. | .. |
| 3′-Methyl-4-methylamino- | +++ | fj | .. | .. |
| 4′-Methyl-4-methylamino- | + | f | .. | .. |
| 2:2′:4′:5-Tetramethyl-4-amino- | o | a | .. | .. |
| 2′:3′:4′:5′:6′-Pentamethyl-4-amino- | o | d | .. | .. |
| §2′:3-Dimethyl- | o[2] | a | ± | ah |
| 4-Chloro-2′:3-dimethyl- | .. | .. | o | a |
| 4-Hydroxy-2′:3-dimethyl- | o[2] | a | ± | h |
| 4-Acetoxy-2′:3-dimethyl- | o | a | .. | .. |
| ¶4-Amino-2′:3-dimethyl- | + | ak | ++ | ahklm |
| 4-Acetylamino-2′:3-dimethyl- | + | a | .. | .. |
| 4-Diacetylamino-2′:3-dimethyl- | + | a | .. | .. |

*, i.e., *p*-aminoazobenzene. †, i.e., *p*-dimethylaminoazobenzene. §, i.e., 2:3′-dimethylazobenzene or 2:3′-azotoluene. ¶, i.e., *o*-aminoazotoluene.

[1], Papillomas of the stomach. [2], Papillomas of the bladdder.

a, Hartwell, 1941. b, Kirby, 1944 ; 1947 ; Kirby and Peacock, 1947. c, Miller and Baumann, 1945*b*. d, Sugiura, Halter, Kensler and Rhoads, 1945. e, Giese, Miller and Baumann, 1945. f, Sugiura, 1948. g, Kinosita, 1937, h, Law, 1941. i, Kirby, 1945. j, Miller and Miller, 1948. k, Crabtree, 1949. l, Shear, 1937. m, Andervont, 1947.

The effect of substituents on the 4-dimethylaminoazobenzene molecule is considered in the next section ; but reference may be made at this stage to the effect of substituents on 4-methylaminoazobenzene. The 2′- and 4′-methyl-derivatives are *less* effective than the parent amine ; but the 3′- derivative is a potent carcinogen (Sugiura, 1945).

(iii) *Substituted 4-dimethylaminoazobenzenes.*

The pronounced activity of 4-dimethylaminoazobenzenes on the livers of rats has led to the preparation and testing of numerous derivatives of this compound (Table IV). These derivatives fall into two main classes.

TABLE IV.—*Substituted 4-dimethylaminoazobenzenes.*

| Compound. | Carcinogenic activity. Rats. | References. |
|---|---|---|
| 4-Dimethylaminoazobenzene | ++ | ab |
| 2-Methyl- | o | bd |
| 3-Methyl- | o | ab |
| 2-Hydroxy- | o | b |
| 2-Fluoro- | +++ | e |
| 2′-Hydroxy- | o | b |
| 2′-Methyl- | + | bd |
| 2′-Nitro- | + | ab |
| 2′-Cloro- | + | ab |
| 2′-Fluoro- | ++ | b |
| 2′-Trifluoromethyl- | o | b |
| *2′-Carboxy- | + | c |
| 3′-Hydroxy- | o | b |
| 3′-Methyl- | +++ | abd |
| 3′-Nitro- | ++ | ab |
| 3′-Chloro- | ++ | ab |
| 3′-Fluror- | +++ | b |
| 3′-Trifluoromethyl- | o | b |
| 3′-Ethoxy- | ± | a |
| 4′-Hydroxy- | o | b |
| 4′-Methyl- | ± | bd |
| 4′-Nitro- | o | b |
| 4′-Chloro- | + | ab |
| 4′-Fluoro- | +++ | b |
| 4′-Trifluoromethyl- | o | b |
| 4′-Arsonic acid | o | c |
| †4′-Sulphonic acid, sodium salt | o | c |
| 2′:4′-Difluoro- | +++ | e |
| 2′:4′:6′-Trifluoro- | +++ | e |
| 2′:4′:6′-Trichloro- | o | b |
| 2′:4′:6′-Tribromo- | o | b |
| 3′:5′-Dimethyl- | o | a |
| 2′:5-Dimethyl- | o | a |
| 2′:4′-Dimethyl- | o | a |

* Methyl red. †, Methyl orange.
a, Miller and Miller, 1948. b, Miller, Sapp and Miller, 1949. c, Hartwell, 1941. d, Miller and Baumann, 1945*b*. e, Miller, Miller and Sapp, 1951.

In the first, the additional substituent is on the same ring as the dimethylamino- group. Only a few such compounds have been tested, but, with the exception of the 2-fluoro- compound, all have been found to be inactive.

In the second class the additional substituent is attached to the other benzene ring. With the exception of the 2'-hydroxy- and 2'-trifluoromethyl- derivatives which are entirely inactive, the *ortho*- or 2'- substituted 4-dimethylaminoazobenzenes are all less active than the parent compound. Only the 2'-fluoro- derivative has about the same activity.

Among the *meta*- or 3'- substituted compounds, the hydroxy- and trifluoromethyl- derivatives are again inactive ; the ethoxy- derivative has trace activity ; the nitro- and chloro- derivatives have approximately the same activity as the parent amine ; and the 3'-methyl- and 3'-fluoro- derivatives are considerably more effective in producing liver tumours than the unsubstituted 4-dimethylaminoazobenzene.

The *para*- or 4'- substituted derivatives are mostly inactive, or only very slightly active ; but the 4'-fluoro- derivative is more effective than 4-dimethylaminoazobenzene itself. The 4'-methyl- and 4'-chloro- compounds are only slightly active, and the 4'-hydroxy-, 4'-nitro-, 4'-trifluoromethyl- and 4'-sulphonic acid derivatives are all completely inactive.

Relatively few di- and tri- substituted derivatives have been tested. It is of some interest, however, that the 3':5'-dimethyl- derivative seems to be inactive (in spite of the fact that *mono*-methyl substituuion at these positions *increases* the potency). Other dimethyl- derivatives are also inactive ; so are the 2':4':6-trichloro- and 2':4':6'-tribomo- compounds.

(iv) *Phenylazonaphthalenes and azonaphthalenes.*

Many commercial dyestuffs and food-colouring matters fall into this group. Many of these are sulphonic acid derivatives, which are probably excreted too rapidly to be effective as carcinogens ; but some are simple hydroxy- or amino- derivatives. Among these, special interest attaches to 1-benzeneazo-2-naphthol (XIX), which is a food-colouring matter and which Kirby and Peacock (1949) have shown to be a liver carcinogen in mice. Very closely related to this substance are Sudan II, Sudan III, and scarlet red, substances which were used in early experiments and shown to promote the healing of wound tissue.

(XIX)

(XX)

(XXI)

It is also noteworthy that the 1- and 2-naphthalene analogues of 4-dimethylaminoazobenzene, namely 4-dimethylaminophenylazo-1′-naphthalene (XX) and 4-dimethylaminophenylazo-2′-naphthalene (XXI), are inactive in rats (Kinosita, 1940; Miller and Baumann, 1945*b*).

It is, however, the unsubstituted azonaphthalenes which form the most interesting series in this group. These compounds were originally examined because operatives engaged in the manufacture of naphthylamine are particularly liable to acquire cancer of the bladder. It was thought that azonaphthalenes might arise by mild oxidation of the naphthylamines, and might conceivably be present as impurities in the commercial product.

2:2′-Azonaphthalene (VI) was found to produce liver tumours in a very large proportion of the mice to which it was administered either by mouth, by subcutaneous injection, or even by painting on the skin. The tumours were mostly cholangioma, but some were hepatoma. A few tumours were also obtained with 1:1′-azonaphthalene (VII); but 1:2′-azonaphthalene (VIII) was found to be inactive (Cook, Hewett, Kennaway and Kennaway, 1940).

TABLE V.—*Phenylazonaphthalenes and Azonaphthalenes.*

| Compound. | | Carcinogenic activity. | | | |
|---|---|---|---|---|---|
| | | Rats. | References. | Mice. | References. |
| Sudan I ("oil orange E"), |  | o | a | + | b |
| Sudan II, |  | o | a | | |
| Sudan III, |  | o | a | | |
| Scarlet red, |  | o | a | | |
| Yellow AB, |  | .. | .. | o | c |
| Yellow OB, |  | .. | .. | o | c |

TABLE V.—(*cont.*)

| Compound. | | Carcinogenic activity. Rats. | References. | Mice. | References. |
|---|---|---|---|---|---|
|  | | o | de | | |
|  | | o | e | | |
|  | αα′ | .. | .. | ± | f |
|  | αβ′ | .. | .. | o | |
|  | ββ′ | .. | .. | +++ | |
|  | (αβ′) | .. | .. | o | f |
|  (Pigment Bordeaux N) | | o | g | | |

a, Hartwell, 1941. b, Kirby and Peacock, 1949. c, Badger, Cook, Hewett, Kennaway, Kennaway and Martin, 1942. d, Miller and Baumann, 1945*b*. e, Kinosita, 1940. f, Cook, Hewett, Kennaway and Kennaway, 1940. g, Maruya, 1938.

(v) *The influence of the azo-linkage.*

This can be studied in two ways : firstly by examining the effect of oxidation at the azo-linkage ; and secondly by progressive replacement of the nitrogen atoms by CH groups.

As to the first method, little has been done. It does appear, however, that 4-amino-2′:3-dimethylazoxybenzene (XXII), the azoxy- compound corresponding to *o*-aminoazotoluene, is inactive in rats (Nagao, 1939). Azoxybenzene and *o*-azoxytoluene (XXIII) are also inactive (Hartwell, 1941).

(XXII)

(XXIII)

The replacement of the nitrogen atoms by CH produces some curious results (Table VI). *N*, *N*-Dimethyl-*N'*-benzal-*p*-phenylenediamine (XXIV) and 4-dimethylaminobenzalaniline (XXV) are inactive in rats (Miller and Baumann, 1945b), but 4-dimethylaminostilbene (XXVI) has been shown to be a potent carcinogen. In mice, however, although tumours were produced, it proved to be much less potent. Several other compounds of the same type, including 2'-methyl-4-dimethylaminostilbene and $\alpha$-(4-dimethylaminophenyl)-$\beta$-(1'-naphthyl)-ethylene are also potent carcinogens for the rat. Apart from sarcomas at the site of injection, these compounds produced many cancers at a distance, including carcinoma of the eyelid, cholangiomata of the liver, multiple mammary fibroadenomata in females, adenomata of the lung, and so on (Haddow, Harris, Kon and Roe, 1948).

(XXIV)

(XXV)

(XXVI)

XXVII

TABLE VI.—*The Influence of the Azo-linkage.*

| Compound. | Carcinogenic activity. Rats. | References. | Mice. | References. |
|---|---|---|---|---|
|  . . . . | ++ | a | + | c |
|  . . . . | o | a | | |
|  . . . . | o | a | | |
|  . . . . | (++) | b | (+) | b |

a, Miller and Baumann, 1945*b*. b, Haddow, Harris, Kon and Roe, 1948. c, Table III.

It seems therefore that the replacement of the azo-linkage in 4-dimethylamino-azobenzene produces a compound having less specialised carcinogenic activity.

On the other hand, it is noteworthy that 2:2′-dinaphthylethylene (XXVII) is completely inactive, in mice, in contrast to the striking activity of the corresponding azonaphthalene towards mice livers (Badger, Cook, Hewett, Kennaway, Kennaway and Martin, 1942).

### *The Effect of Diet.*

It is not proposed to give a detailed review of the effect of diet on the carcinogenic activity of the azo- compounds ; but a brief account is necessary (for a comprehensive review see Rusch, Baumann, Miller and Kline, 1945).

Nearly all the early work in this field was carried out in Japanese laboratories. The usual procedure was to add a solution of the azo- compound in olive oil to a measured quantity of ground rice. The rats were then fed this mixture, which was supplemented with a little carrot. Using this deficient diet, 4-dimethylaminoazobenzene was found to induce liver tumours in a high percentage of the rats used (Kinosita, 1937).

It soon transpired that the diet plays a very important role in this type of carcinogenesis. With more balanced diets the appearance of liver tumours was either delayed, or entirely prevented. The use of either wheat, rye or millet in place of rice afforded some protection ; and various substances added to the basal diet were found to reduce the incidence of liver tumours to a marked degree. Beef liver and yeast were found to be especially effective in this respect (Nakahara, Mori and Fujiwara, 1939 ; Ando, 1938 ; Sugiura and Rhoads, 1941).

These observations indicated that vitamins of the B group might be involved, and tests have shown that while biotin, pyridoxine and vitamin $B_{12}$ increase the incidence of liver tumours (du Vigneaud, Spangler, Burk, Kensler, Sugiura and Rhoads, 1942 ; Miner, Miller, Baumann and Rusch, 1943 ; Day, Payne and Dinning, 1950), riboflavin has a marked protective action. This protective action is greatest when the diet also contains adequate protein ; nearly all the so-called protective diets are rich in both riboflavin and protein (Kensler, Sugiura, Young, Halter and Rhoads, 1941 ; Miner, Miller, Baumann and Rusch, 1943).

The protective action seems to be closely associated with the concentration of riboflavin in the liver. Rats fed diets known to accelerate tumour induction were found to have *less* riboflavin in the livers than controls ; and rats fed various protective diets were found to have *greater* hepatic riboflavin levels than controls (Miller, Miller, Kline and Rusch, 1948). Moreover, certain azo- compounds markedly lower the hepatic concentration of riboflavin (Kensler, Sugiura and Rhoads, 1940). The decrease appears to be roughly equivalent to the carcinogenicity of the dye. Thus 3′-methyl-4-dimethylaminoazobenzene was most effective, 4-dimethylaminoazobenzene and 4-methylaminoazobenzene were fairly effective, and 2′-methyl-4-dimethylaminoazobenzene, 4′-methyl-4-dimethylaminoazobenzene, *o*-aminoazotoluene and 4-aminoazobenzene and azobenzene had little or no effect (Griffin and Baumann, 1946). Griffin and Baumann (1948) conclude that there is an inverse relationship between the rate of tumour development and the level of hepatic riboflavin maintained on any particular dietary regimen. Miller and Miller (1947) have found that when azo- compounds are fed to rats, a portion of the dye becomes very tightly bound in the liver. Especially significant was the observation that the amount of bound azo- compound found in the livers of rats fed 4-dimethylaminoazobenzene and 4-methylaminoazobenzene on a low riboflavin diet was greater than that in rats fed on a high riboflavin diet. Furthermore, the potent carcinogenic compounds gave higher levels of bound azo-dye, in a shorter time than the less active compounds (Miller, Miller, Sapp and Weber, 1949).

In addition, Silverstone (1948) has shown that there is a direct relationship (Table VII) between the level of carcinogenic dye (in this case 4-dimethylamino-azobenzene) in the liver, and the incidence of liver tumours.

TABLE VII.—*Diet and Tumour Incidence.*

| Diet. | Average level of carcinogenic azo-dye/g. of liver at 8 weeks' period. | Per cent incidence hepatomas at 6 months. |
|---|---|---|
| High protein, high fat | 0·32 μg. | 25 |
| Brown rice, yeast | 0·22 ,, | 37 |
| Low protein, high fat | 0·46 ,, | 58 |
| Low protein, low fat | 0·51 ,, | 71 |
| Brown rice | 0·89 ,, | 96 |

It is reasonable to conclude therefore (i) that the greater the concentration the carcinogen can achieve in the liver, the greater its carcinogenic activity; and (ii) that a high riboflavin content in the liver reduces the concentration of carcinogen in that organ and hence reduces the tumour incidence.

The effect of the riboflavin may be to hinder the conversion of the azo-compound into its carcinogenic derivative (possibly a protein-azo- compound complex); or the riboflavin may increase the ability of the liver to detoxify the azo- compound by converting it into simpler non-carcinogenic compounds.

### *Metabolism of Azo- Compounds.*

The carcinogenic azo- compounds do not, as a rule, produce tumours at the site of application in rats, but appear to have a more or less specific action on the liver. Some of the compounds do produce tumours in the urinary bladder and tumours at some other sites have also been observed. For example, Lowenhaupt (1949) observed some lymphoblastic lymphosarcomas in rats bearing intrasplenic pellets of 4-dimethylaminoazobenzene. Hoch-Ligeti (1949) obtained primary pancreatic tumours in rats fed 4-dimethylaminoazobenzene, and it seems that the dye can affect other organs if the liver is " protected " by a suitable diet. Nevertheless, the specificity is very striking and is in marked contrast to the carcinogenic polycyclic hydrocarbons such as benzpyrene and methylcholanthrene, which produce tumours at the site of application.

For this reason the metabolism of the azo- compounds is of considerable importance, and it has often been suggested that the azo- compounds are not carcinogenic *per se*, but that they are converted into active substances in the liver.

Most of the work in this field has been carried out with 4-dimethylaminoazo-benzene. This undergoes demethylation in the rat, relatively large amounts of 4-methylaminoazobenzene and 4-aminoazobenzene being recoverable (Kensler, Magill and Sugiura, 1947). Dealkylation of the diethyl- derivative also occurs (Kensler, Magill, Sugiura and Rhoads, 1946). It is also noteworthy that when 4-methylaminoazobenzene is administered, the liver has been found to contain

4-aminoazobenzene and 4-dimethylaminoazobenzene as well as the mono-methyl compound. On the other hand, when 4-aminoazobenzene was fed, this was the only dye found in the tissues (Miller, Miller and Baumann, 1945). Special methods for the determination of these compounds have been devised by Miller and Baumann (1945*a*).

4-Dimethylaminoazobenzene also undergoes reductive fission in rats, and *p*-phenylenediamine, *p*-aminophenol and their acetylation products have been detected in the urine (Stevenson, Dobriner and Rhoads, 1942). *p*-Phenylenediamine and *N*, *N*-dimethyl-*p*-phenylenediamine have been shown to inhibit certain important enzyme systems, and Kensler, Dexter and Rhoads (1942) therefore suggested that the carcinogenic activity may be due to " split products " of these types (or to a semi-quinone free radical of similar structure). In the opinion of the present reviewer this interesting hypothesis has now been proved erroneous. *p*-Phenylenediamine and its *N*, *N*-dimethyl- derivative have both been shown to be devoid of carcinogenic activity. Moreover, there are many azo- compounds which would be expected to yield these diamines on reductive fission, but which are entirely inactive or have only slight activity. For example, 4′-methyl-4-dimethylaminoazobenzene is much less active than 4-dimethylaminoazobenzene as a carcinogen, yet both would be expected to yield the same diamines. Similarly, it is surprising that 1′-(4′-dimethylaminophenylazo)naphthalene and 2′-(4-dimethylaminophenylazo)naphthalene are both entirely inactive (Table V). Many other examples could be given (Kirby, 1945; Crabtree, 1949; Rusch, Baumann, Miller and Kline, 1945). It is also significant that an amino- substituent is not essential for activity in an azo- compound, 2:2′-azonaphthalene being a striking example of an active carcinogen lacking such a group.

Another interesting hypothesis was put forward by Cook, Hewett, Kennaway and Kennaway (1940). It was suggested that the azo- compound may first be reduced to the hydrazo- derivative, and subsequently suffer the benzidine transformation and loss of ammonia to form a carbazole derivative. In particular, in the case of 2:2′-azonaphthalene (XXVIII) it was suggested that the true carcinogen, formed in the liver, is 3:4:5:6-dibenzcarbazole (XXXI). This transformation, illustrated by the scheme (XXVIII)⟶(XXXI), can certainly be carried out very readily *in vitro*, and although there is no proof that it does occur *in vivo*, certain facts are suggestive. It has been shown, for example, that the postulated intermediate diamine (XXX) produces liver tumours of the cholangiomatous type

(as does the azo-compound) ; and 3:4:5:6-dibenzcarbazole has not only been shown to produce liver tumours but also tumours at the site of application (Boyland and Brues, 1937).

Evidence of a negative nature is provided by the related 1:2′-azonaphthalene and its transformation products. In this case the azo- compound, and the diamine derived from it, have little or no action on the liver. This diamine yields 1:2:5:6-dibenzcarbazole by prolonged boiling with hydrochloric acid, and this appears to be almost without carcinogenic action on the liver (Badger, Cook, Hewett, Kennaway, Kennaway and Martin, 1942).

Furthermore, Elson and Warren (1944) have found that when azobenzene is administered to rats, the urine contains not only aniline, but also a water-soluble compound (probably a derivative of hydrazobenzene) which on treatment with dilute acid is converted into benzidine. With 4-dimethylaminoazobenzene it seems that a benzidine type of rearrangement may take place *in vivo* even more readily than with azobenzene.

In spite of these successes, it is unlikely that this is the full story. Benzidine rearrangement of the hydrazo- compound (XXXII) resulting from 4-dimethylaminoazobenzene is known to give 2:4′-diamino-5-dimethylaminodiphenyl (XXXIII) together with some semidine (XXXIV) (Jacobsen, 1922) ; and the former has been shown to be inactive even when fed at a high level for 10 months. (In this connection, however, it is worth remembering that 4-dimethylaminodiphenyl produces cancer in a variety of organs in the rat (Miller, Miller, Sandin and Brown, 1949).

(XXXII) (XXXIII) (XXXIV)

The theory is not obviously applicable to other substituted azo- compounds, especially as even closely related compounds often give different types of products in the benzidine reaction. 4-Aminoazobenzene, for example, unlike the 4-dimethylamino- derivative, gives the *p*-semidine as the major product (Jacobsen, 1922). Furthermore, Miller, Miller and Sapp (1951) have found that carcinogenic activity can be demonstrated in various substituted compounds which are unlikely to undergo the rearrangement. Only one of the five possible benzidine or semidine rearrangements could possibly occur in the case of 2′:4′:6′-trifluoro-4-dimethylaminoazobenzene (which is more potent than the parent amine) ; and this lone possibility involves a semidine rearrangement to the 2-position. The high activity of the 2-fluoro- derivative makes this possibility very unlikely. Miller, Miller and Sapp (1951) therefore maintain that although such rearrangements may occur in the metabolism of 4-dimethylaminoazobenzene, it is doubtful if they play any decisive role in carcinogenesis by this dye.

*Chemical Constitution and Carcinogenic Activity.*

Some azo- compounds are known to exist both in *cis* and *trans* forms ; but the *trans* form is always the more stable. All the compounds which have been tested for carcinogenic activity appear to have been *trans* compounds, so that this is the only configuration which need be considered here.

The structure of *trans* azobenzene itself has been established by analysis of the X-ray diffraction pattern (de Lange, Robertson and Woodward, 1939). It is a flat molecule, and has the dimensions shown in the accompanying diagram (XXXV). The C -- N bonds (1·41 Å) are somewhat shorter than normal single bonds of this type (1·47 Å) ; and the N=N bond (1·23 Å) is somewhat longer than a normal double bond of this nature (1·20 Å). The explanation is, of course, that the azo- group is conjugated with the aromatic rings. In other words, structures of type (XXXVI) contribute to the " resonance hybrid". The C — N bonds therefore have some double bond character, and hence are shorter than pure single bonds. Similarly, the N=N bond has some single bond character, and hence is longer than a pure double bond of this nature. In related compounds e.g., azonaphthalenes) the character of the N=N bond is dependent on the extent of conjugation with the aromatic ring systems ; the greater the conjugation, the smaller the double bond character of the N=N bond.

(XXXV)

(XXXVI)

It must be remembered that each nitrogen atom has a " lone pair " of electrons not involved in bond formation. These electrons are also shared with the ring systems to some extent, so that azo- nitrogen atoms are relatively non-basic.

There is thus a certain electron density associated with the N=N linkage in azo- compounds, this density being controlled by the extent of conjugation with the particular aromatic ring systems involved.

Substituents can also affect this density of electrons, and it is instructive to consider this aspect of the matter in some detail.

Substituents can be expected to modify the electron density at the N=N linkage in two ways. In the first place they may modify the extent of conjugation of the azo- group with the ring systems, and either increase or decrease the double bond character. It seems likely, however, that such modifications are of a minor character, and that the electron density will be affected to a greater degree by direct transfer of charge to (or from) one of the nitrogen atoms.

An electron-donating substituent (A) in the *ortho* or *para* position, for example, would increase the electron-density around the nitrogen further removed from it, as in structure (XXXVII). Similarly, an electron-attracting substituent (B) in the *ortho* or *para* position would decrease the density of electrons around the same nitrogen atom, as in structure (XXXVIII).

This is equivalent to saying that structures such as (XXXVIIa) and (XXXVIIIa) contribute to the resonance hybrids of such substituted compounds to a significant extent.

(XXXVII)

(XXXVIII)

(XXXVIIa)

(XXXVIIIa)

Substituents in the *meta* positions, however, are not conjugated with the azo- group, and hence can affect the electron density only by virtue of their inductive effects. In this case, the *nearer* nitrogen atom will be more affected, as in structure (XXXIX).

(XXXIX)

These effects of substituents are of importance because Pullman (1946 ; see also Pullman and Pullman, 1946) has suggested that carcinogenic activity in this type of compound is to be associated with a certain critical density of electrons on the azo-linkage (called the K′ position). This hypothesis is analogous to that for the polycyclic aromatic hydrocarbons, which associates carcinogenic activity with a critical electron density on the phenanthrene-type double bond, called the K position (Pullman and Pullman, 1946 ; Pullman, 1947 ; Badger, 1948).

Pullman (1947) divides the azo- compounds into three groups :

(*a*) Those compounds which are inactive or only slightly active by virtue of an insufficient electron density on the K′ region. This group includes compounds such as the anils (XL and XLI), 4-aminoazobenzene (XLII), and 4-diethylamino-azobenzene (XLIII).

(XL)

(XLI)

(XLII)

(XLIII)

(*b*) This group includes the active compounds, that is, those substances having an electron density at the K′ position within the postulated upper and lower limits. Some examples are *o*-aminoazotoluene (XLIV), 4-dimethylamino-azobenzene (XLV), and 3′-methyl-4-dimethylaminoazobenzene (XLVI).

(XLIV)

(XLV)

(XLVI)

(*c*) The third group includes those which are only slightly active or inactive by virtue of their excessive electron density at the K′ position. Derivatives of 4-dimethylaminoazobenzene with a methyl group in one of the *ortho* or *para* positions (e.g., XLVII, XLVIII and XLIX) fall into this class.

(XLVII)

(XLVIII)

(XLIX)

Unfortunately the available data at present are too few for a really satisfactory examination of this interesting hypothesis. Very few azo- compounds having electron-donating substituents other than alkylamino- groups have yet been examined ; and the testing of a further series of unsubstituted compounds such as the phenylazonaphthalenes, azophenanthrenes, and so on, would also be of value.

The evaluation of the electron density at the K′ position by experimental methods is also necessary before any satisfactory comparison between electron density and carcinogenic activity can be made.

Hamon (1947) has studied the rates of hydrogenation of various azo-compounds ; but unfortunately this is not a very satisfactory method for the determination of electron densities at the K′ position. As she pointed out, every substituent in the azobenzene molecule, besides having an effect on the benzene ring, the other substituents, and on the azo-linkage, can also have an effect on the catalyst (in this case Raney nickel). In addition, there is the possibility that the reduction products of the different azo- compounds may poison the catalyst to varying degrees, and this retarding influence on the rate of reduction may vary from one azo- compound to another.

In a recent attempt to assess the influence of substituents, Rogers, Campbell and Maatman (1951) have determined the ionisation constants of some 4′-substituted 4-dimethylaminoazobenzenes. It seems that the first proton is added to an azo-nitrogen and the second to the dimethylamino-nitrogen. The first and second pKa values were determined, and the results indicate that the greater the electron-donating power of the 4′-substituent, the more easily is the proton added

(i.e., the stronger the basicity of the compound). The effect of the 4′-substituents on the proton affinity of the azo-nitrogen atoms was found to be in the same order as the net electron affinities of the groups as measured by Hammett's substituent constant, " σ " (Hammett, 1940).

This means that in a series of azobenzenes, the electron densities at the K′ position must be directly related to the substituent constants of the substituents. It should be possible, therefore, to examine the validity of the Pullman hypothesis by comparing the carcinogenic activities of, say, a series of 3′- and 4′- substituted 4-dimethylaminoazobenzenes with the substituent constants of the substituents. This is done in Table VIII, where the compounds are arranged in order of increasing basicity (increasing proton affinity). Too few compounds of this nature have been tested for any supposed correlation to be of significance ; but at least it may be said that the available results do support the suggestion that activity is associated with an optimum electron density (or proton affinity) at the K′ position. The 3′-ethoxy- and 4′-chloro- compounds are less active than might be predicted, but otherwise the correlation is as good as could be expected. (Of course, many other factors such as the rate of metabolic detoxication, etc. etc., may also be involved.)

It is not possible to extend this treatment to the 2′- or 2-substituted compounds as steric factors are of importance with such "*ortho*" derivatives, and no σ values can be determined. Similarly, substituents in the 3-position would be expected to interfere sterically with the dimethylamino- group, and such compounds cannot therefore provide any information of value for the present purpose.

TABLE VIII.—*Substituent Constants and Carcinogenic Activities of some 4′-substituted 4-Dimethylaminoazobenzenes.*

| Substituent. | Substituent constant, σ. | Carcinogenic activity (rats). |
|---|---|---|
| 4′-Nitro- . | +·778 | o |
| 3′-Nitro- . | +·710 | ++ |
| 3′-Chloro- | +·373 | ++ |
| 3′-Fluoro- | +·337 | +++ |
| 4′-Chloro- | +·227 | + |
| 3′-Ethoxy- | +·15 | ± |
| 4′-Fluoro- | +·062 | +++ |
| Parent compound | ·000 | ++ |
| 3′-Methyl- | −·069 | +++ |
| 3′:5′-Dimethyl- | −·138 | o |
| 4′-Methyl- | −·170 | ± |

It should also be possible to examine the validity of the Pullman hypothesis by studying the rate of addition of electrophilic reagents to the K′ region in a series of carcinogenic and related non-carcinogenic azo- compounds. Other things being equal, the greater the electron density at the nitrogen atoms, the greater the reactivity towards electrophilic reagents. The peracids are known to be electrophilic reagents (Swern, 1947), and perbenzoic acid has therefore been used against a series of substituted azobenzenes, and azonaphthalenes Badger and Lewis, 1951 ; Badger and Lewis, 1952, unpublished results). The reaction was shown to be bimolecular and may therefore be represented by the equation :

$$R{-}N{=}N{-}R + Ph.CO_3H \longrightarrow R{-}\underset{\substack{\downarrow\\ O}}{N}{=}N{-}R + Ph.CO_2H$$

Azobenzene, for example, gave azoxybenzene, and azonaphthalenes gave azoxynaphthalenes.

Among the non-substituted compounds, the different reaction rates must depend on the fact that the azo- group is not always conjugated with an aromatic ring to the same extent. As expected, and in agreement with the Pullman hypothesis, electron-releasing subtituents increase the reaction rate, and electron-attracting substituents retard the reaction (Table IX). For example, 4-methoxyazobenzene reacts much more rapidly with perbenzoic acid than azobenzene, and 4-carbethoxyazobenzene reacts more slowly than the unsubstituted compound. Halogenated azobenzenes also react more slowly than azobenzene itself, the inhibitory effect of a halogen in the 4- position being Cl>Br>F.

TABLE IX.—*Rates of Reaction of Perbenzoic Acid with Azobenzene and Related Compounds, at 25°.*

| Compound. | Reaction rate $10^3 k_2^{25°}$. |
|---|---|
| Azobenzene | 13·9 |
| 4-Fluoroazobenzene | 9·7 |
| 4-Chloroazobenzene | 7·5 |
| 4-Bromoazobenzene | 9·2 |
| 4-Methoxyazobenzene | 58 |
| 1-Phenylazonaphthalene | 14·5 |
| 2-Phenylazonaphthalene | 14·5 |
| 1:1′-Azonaphthalene | 2·5 |
| 1:2′-Azonaphthalene | 17·7 |
| 2:2′-Azonaphthalene | 17·6 |

In conclusion, although it seems impossible at present to come to any very definite decisions, recent work has been encouraging, and there is little doubt that future research will yield significant results on the relationship between chemical constitution and carcinogenic activity in this class of compound.

### SUMMARY.

1. Many derivatives of azobenzene produce cancer of the liver when administered to rats and mice on a restricted diet.

2. The diet must be low in riboflavin and in protein.

3. Most of the active compounds are amino-, methylamino- or dimethylamino-derivatives of azobenzenes ; but an amino- group is not essential for activity of this nature (e.g., 2:2′-azonaphthalene).

4. Additional substituents have variable effects depending on their nature and position.

5. The hypothesis that carcinogenic activity in this series is associated with an optimum density of electrons at the —N=N— linkage is supported by recent experimental work ; but further research is necessary before any definite conclusions can be drawn.

### REFERENCES.

ANDERVONT, H. B.—(1947) *J. nat. Cancer Inst.*, **7**, 431.
ANDO, T.—(1938) *Gann*, **32**, 252.
BADGER, G. M.—(1948) *Brit. J. Cancer*, **2**, 309.
*Idem*, COOK, J. W., HEWETT, C. L., KENNAWAY, E. L., KENNAWAY, N. M., AND MARTIN, R. H.—(1942) *Proc. Roy. Soc.*, **B**, **131**, 170.

*Idem* AND LEWIS, G. E.—(1951) *Nature*, **167**, 403.
BOYLAND, E., AND BRUES, A. M.—(1937) *Proc. Roy. Soc.*, **B**, **122**, 429.
COOK, J. W.—(1939) *Ergeb. Vitam.- u. Hormonforsch.*, **2**, 213.—(1943) Royal Institute of Chemistry, Lecture.—(1948) *Brit. J. Nutr.*, **1**, 245.
*Idem*, HEWETT, C. L., KENNAWAY, E. L., AND KENNAWAY, N. M.—(1940) *Amer. J. Cancer*, **40**, 62.
*Idem* AND KENNAWAY, E. L.—(1938) *Ibid.*, **33**, 50.
CRABTREE, H. G.—(1949) *Brit. J. Cancer*, **3**, 387.
DAY, P. L., PAYNE, L. D., AND DINNING, J. S.—(1950) *Proc. Soc. exp. Biol. N.Y.*, **74**, 854.
ELSON, L. A., AND WARREN, F. L.—(1944) *Biochem. J.*, **38**, 217.
FISCHER, B.—(1906) *Münch. med. Wschr.*, **53**, 2041.
GIESE, J. E., MILLER, J. A., AND BAUMANN, C. A.—(1945) *Cancer Res.*, **5**, 337.
GRIFFIN, A. C., AND BAUMANN, C. A.—(1946) *Arch. Biochem.*, **11**, 467.—(1948) *Cancer Res.*, **8**, 279.
HADDOW, A., HARRIS, R. J. C., KON, G. A. R., AND ROE, E. M. F.—(1948) *Phil. Trans.*, **241**, 147.
HAMMETT, L. P.—(1940) 'Physical Organic Chemistry.' New York (McGraw-Hill), p. 184.
HAMON, V.—(1947) *Ann. Chim.*, **2**, 233.
HARTWELL, J. L.—(1941) 'Survey of Compounds which have been Tested for Carcinogenic Activity.' National Cancer Institute, U.S. Public Health Service.
HAYWARD, E.—(1909) *Münch. med. Wschr.*, **56**, 1836.
HOCH-LIGETI, C.—(1949) *Brit. J. Cancer*, **3**, 285.
JACOBSON, P.—(1922) *Liebigs Ann.*, **428**, 76.
KENSLER, C. J., DEXTER, S. O., AND RHOADS, C. P.—(1942) *Cancer Res.*, **2**, 1.
*Idem*, MAGILL, J. W., AND SUGIURA, K.—(1947) *Ibid.*, **7**, 95.
*Iidem* AND RHOADS, C. P.—(1946) *Arch. Biochem.*, **11**, 376.
*Idem*, SUGIURA, K., AND RHOADS, C. P.—(1940) *Science*, **91**, 623.
*Idem*, SUGIURA, K., YOUNG, N. F., HALTER, C. R., AND RHOADS, C. P.—(1941) *Ibid.*, **93**, 308.
KINOSITA, R.—(1937) *Trans. Soc. Path. Jap.*, **27**, 665.—(1940) *Gann*, **34**, 165.
KIRBY, A. H. M.—(1944) *Nature*, **154**, 668.—(1945) *Cancer Res.*, **5**, 683.—(1947) *Ibid.*, **7**, 333.
*Idem* AND PEACOCK, P. R.—(1947) *J. Path. Bact.*, **59**, 1.—(1949) *Glasg. med. J.*, **30**, 364.
LANGE, J. J. DE, ROBERTSON, J. M., AND WOODWARD, I.—(1939) *Proc. Roy. Soc.*, **B**, **171**, 398.
LAW, L. W.—(1941) *Cancer Res.*, **1**, 397.
LOWENHAUPT, E.—(1949) *Ibid.*, **9**, 121.
MARUYA, H.—(1938) *Trans. Soc. Path. Jap.*, **28**, 541.
MILLER, E. C., AND MILLER, J. A.—(1947) *Cancer Res.*, **7**, 468.
*Iidem*, KLINE, B. E., AND RUSCH, H. P.—(1948) *J. exp. Med.*, **88**, 89.
*Idem*, MILLER, J. A., SANDIN, R. B., AND BROWN, R. K.—(1949) *Cancer Res.*, **9**, 504.
*Idem*, MILLER, J. A., SAPP, R. W., AND WEBER, G. M.—(1949) *Ibid.*, **9**, 336.
MILLER, J. A., AND BAUMANN, C. A.—(1945*a*) *Ibid.*, **5**, 157.—(1945*b*) *Ibid.*, **5**, 227.
*Idem* AND MILLER, E. C.—(1948) *J. exp. Med.*, **87**, 139.
*Iidem* AND BAUMANN, C. A.—(1945) *Cancer Res.*, **5**, 162.
*Idem*, MILLER, E. C., AND SAPP, R. W.—(1951) *Ibid.*, **11**, 269.
*Idem*, SAPP, R. W., AND MILLER, C. E.—(1949) *Ibid.*, **9**, 652.
MINER, D. L., MILLER, J. A., BAUMANN, C. A., AND RUSCH, H. P.—(1943) *Ibid.*, **3**, 296.
NAGAO, N.—(1939) *Proc. imp. Acad. Japan*, **15**, 321.
NAKAHARA, W., MORI, K., AND FUJIWARA, T.—(1939) *Gann*, **33**, 406.

Otsuka, L., and Nagao, N.—(1936) *Ibid.*, **30**, 561.
Pullman, A.—(1947) *Ann. Chim.*, **2**, 5.
*Idem* and Pullman, B.—(1946) *Rev. sci.*, *Paris*, **84**, 145.
Pullman, B.—(1946) *C. R. Acad. Sci.*, *Paris*, **222**, 1501.
Rogers, M. T., Campbell, T. W., and Maatman, R. W.—(1951) *J. Amer. chem. Soc.*, **73**, 5122.
Rusch, H. P., Baumann, C. A., Miller, J. A., and Kline, B. E.—(1945) Amer. Ass. Adv. Sci., 'Research Conference on Cancer,' p. 267.
Sasaki, T., and Yoshida, T.—(1935) *Virchows Archiv.*, **295**, 175.
Shear, M. J.—(1937) *Amer. J. Cancer*, **29**, 269.
Silverstone, H.—(1948) *Cancer Res.*, **8**, 301.
Stevenson, E. S., Dobriner, K., and Rhoads, C. P.—(1942) *Cancer Res.*, **2**, 160.
Stöber, H.—(1909) *Münch. med. Wschr.*, **56**, 129.
Sugiura, K.—(1948) *Cancer Res.*, **8**, 141.
*Idem* and Rhoads, C. P.—(1941) *Ibid.*, **1**, 3.
*Idem*, Halter, C. R., Kensler, C. J., and Rhoads, C. P.—(1945) *Ibid.*, **5**, 235.
Swern, D.—(1947) *J. Amer. chem. Soc.*, **69**, 1692.
Vigneaud, V. du, Spangler, J. M., Burk, D., Kensler, C. J., Sugiura, K., and Rhoads, C. P.—(1942) *Science*, **95**, 174.
Yoshida, T.—(1932) *Proc. imp. Acad. Japan*, **8**, 464.—(1933) *Trans. Soc. Path. Jap.*, **23**, 636.—(1934) *Ibid.*, **24**, 523.—(1935) *Gann*, **29**, 295.